# Human Activity Sensing with Wireless Signals: A Survey

**DOI:** 10.3390/s20041210

**Published:** 2020-02-22

**Authors:** Jiao Liu, Guanlong Teng, Feng Hong

**Affiliations:** Department of Computer Science and Technology, Ocean University of China, Qingdao 266100, China; liujiao@stu.ouc.edu.cn (J.L.); tgl@stu.ouc.edu.cn (G.T.)

**Keywords:** wireless sensing, activity recognition, counting, detection, tracking

## Abstract

Wireless networks have been widely deployed with a high demand for wireless data traffic. The ubiquitous availability of wireless signals brings new opportunities for non-intrusive human activity sensing. To enhance a thorough understanding of existing wireless sensing techniques and provide insights for future directions, this survey conducts a review of the existing research on human activity sensing with wireless signals. We review and compare existing research of wireless human activity sensing from seven perspectives, including the types of wireless signals, theoretical models, signal preprocessing techniques, activity segmentation, feature extraction, classification, and application. With the development and deployment of new wireless technology, there will be more sensing opportunities in human activities. Based on the analysis of existing research, the survey points out seven challenges on wireless human activity sensing research: robustness, non-coexistence of sensing and communications, privacy, multiple user activity sensing, limited sensing range, complex deep learning, and lack of standard datasets. Finally, this survey presents four possible future research trends, including new theoretical models, the coexistence of sensing and communications, awareness of sensing on receivers, and constructing open datasets to enable new wireless sensing opportunities on human activities.

## 1. Introduction

The rapid development and the pervasiveness of wireless networks has stimulated a surge in relevant research of wireless sensing, including detection, recognition, estimation, and tracking of human activities. Wireless sensing reuses the wireless communication infrastructure, so it is easy to deploy and has a low cost. Compared to sensor-based and video-based human activity sensing solutions, wireless sensing is not intrusive and of fewer privacy concerns. Specifically, video-based sensing is restricted in line-of-sight (LoS) and light conditions and raises more privacy concerns. Sensor-based sensing incurs extra cost due to additional sensors, as well as accompanying some inconvenience on wearing for users.

During the propagation of the wireless signal from the transmitter to the receiver, the wireless signal is affected by obstacles in the transmission space, resulting in attenuation, refraction, diffraction, reflection, and multipath effects. Therefore, wireless signals arrived at the receiver carry the environmental information. Human activity will affect wireless signal propagation, which can be captured inside the received signals. Since different activities may lead to various patterns inside wireless signals, it can be used for different wireless sensing applications. Recent research has applied wireless sensing on motion detection, activity recognition, action estimation, and tracking. Various wireless sensing applications target their specific purpose and use unique signal processing techniques and recognition/estimation algorithms. To enhance a thorough understanding of existing wireless sensing techniques and provide insights for future directions, this survey conducts a review of the existing research on human activity sensing with wireless signals.

**Figure 1:**
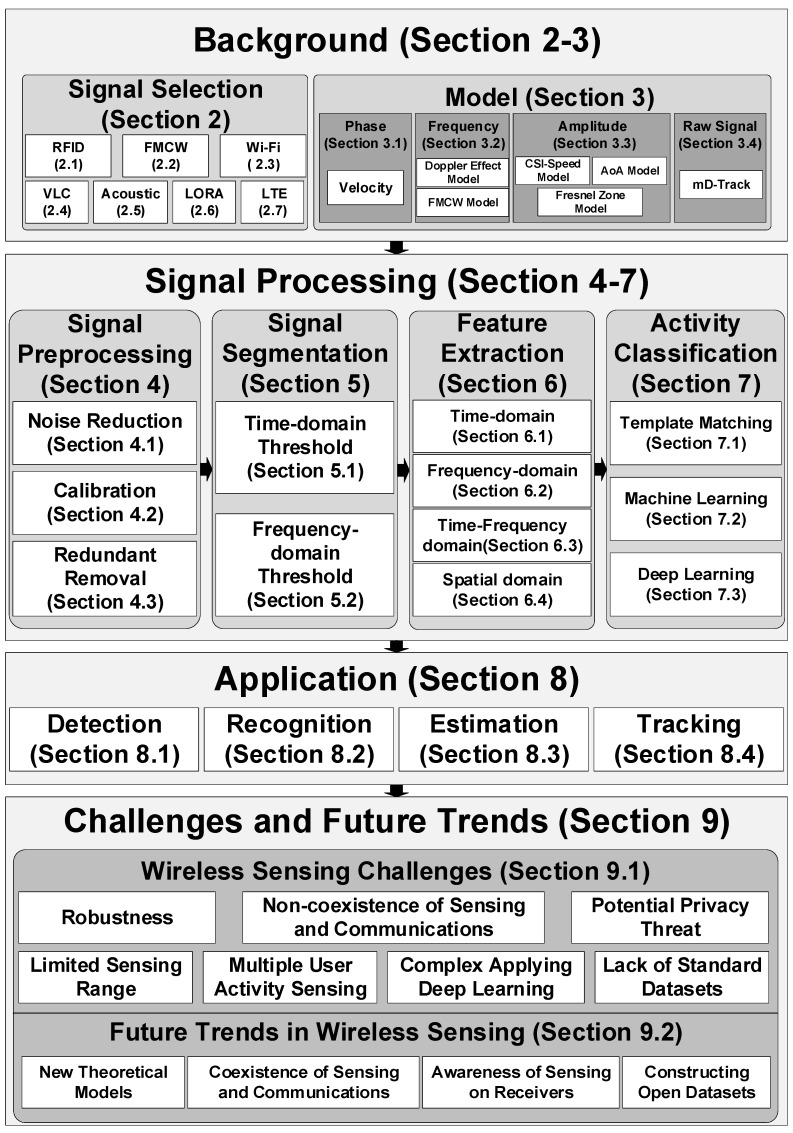
Overview of wireless sensing and survey organization.

Figure 1 shows an overview of the survey. After discussing the related work in Section 2, we introduce the background and characteristics of wireless signals in Section 3. The theoretical models from wireless signals to the features of human motion are discussed in Section 4. The signal preprocessing for noise and outlier reduction are shown in Section 5. The preprocessed signal sequences are fed to the detection module to cut out the signal segment corresponding to every single action, shown in Section 6. The feature extraction applied on the action segment are described in Section 7. The activity classification algorithms are compared in Section 8. According to the output types, different applications of wireless activity sensing are reviewed in Section 9. With the development and deployment of new wireless infrastructure, the challenges and future trends for enabling new sensing applications and capabilities are discussed in Section 10. The main contributions of this survey are as follows.

1. We provide a comprehensive review of human activity sensing with wireless signals from seven perspectives, including wireless signals, theoretical models, signal preprocessing techniques, activity segmentation, feature extraction, classification, and application.
2. We discuss the future trends on human activity sensing with wireless signals, including new theoretical models, the coexistence of sensing and communications, awareness of sensing on receivers, and constructing open datasets.

## 2. Related Works

There are some surveys on wireless sensing with specific wireless signals for specific application scenarios. Some surveys [1,2] focus on indoor localization and tracking with wireless signals. Yang et al. [1] present a survey on the studies of applying Wi-Fi CSI for indoor localization and tracking. Xiao et al. [2] focus on both device-free and device-based localization with multiple kinds of wireless signals.

Many surveys [3,4,5,6,7,8] concentrate on the application of Wi-Fi CSI for human behavior recognition. Zou et al. [3] give a brief review of the Wi-Fi CSI based sensing systems, which describes the concept of Wi-Fi CSI, presents the general framework of Wi-Fi CSI sensing systems, and discusses the remaining challenges and open issues. Wu et al. [4] divide Wi-Fi CSI sensing solutions into two categories of patter-based and model-based and give a brief review on the studies of two approaches and show the potential of Fresnel zone model-based sensing systems. Yousefi et al. [5] present a short survey on human behavior recognition using Wi-Fi CSI, which presents the framework of activity recognition and points out that deep learning techniques may improve the activity recognition performance. In [6], the authors provide a review of recent advances in CSI-based sensing systems, which illustrates the applications of human motion detection, macro and micro activity recognition, and human localization, with discussions of the limitations and challenges. Wang et al. [7] introduce the basic principle of Wi-Fi CSI-based behavior recognition and review the key algorithms for each step of Wi-Fi sensing, including base signal selection, signal pre-processing, feature extraction, and classification. Ma et al. [8] give a comprehensive survey on Wi-Fi sensing, including signal processing techniques, algorithms, applications, challenges, and future trends.

Liu et al. [9] review the existing wireless sensing systems in terms of their basic principles, techniques, and system structures with Wi-Fi RSS and CSI, FMCW, and Doppler shift. In [9], they review the existing studies from the perspectives of specific applications, including intrusion detection, room occupancy monitoring, daily activity recognition, gesture recognition, vital signs monitoring, user identification, and indoor localization. 

This survey differs from previous surveys on three points. Firstly, it expands the wireless signal types for human activity sensing and describes the pros and cons for each type of wireless signal on sensing, which includes RFID, FMCW, Wi-Fi, visible light, acoustic, LoRa, and LTE. Secondly, this survey provides a comprehensive summary of the models between human activity and wireless signals and a detailed comparison on signal pre-processing, signal segmentation, feature extraction, and classification for each existing wireless sensing studies. Thirdly, the survey analyzes the potential challenges and points out future trends to enhance wireless sensing capabilities. Table 1 summarizes the comparison between this survey and previous surveys.

### 2.1. RFID

Radio Frequency Identification (RFID) is a communication technology for contactless two-way communication to identify and exchange data. In general, the RFID system consists mainly of low-cost tags and readers. The tags contain built-in coils and chips. The reader sends out a specific frequency signal. When the tag is close enough to the reader, the coil electromagnetic induction generates electrical energy after the tag receives the transmitted signals, and the chip transmits the stored information through the antennas. The reader accepts and recognizes the information sent by the tag, then delivers the identification results to the host.

The tags can be classified according to internal electrical energy and frequency. On the one hand, tags can be divided into active and passive tags according to whether they can communicate actively with the reader. The difference lies in the availability of internal electrical energy. On the other hand, in terms of frequency, tags can be divided into low frequency, high frequency, UHF, and microwave tags.

As shown in Table 2, passive RFID mainly works between 125 kHz and 13.56 MHz, and its communication distance is very close, usually less than 1.2 m. Active RFID is powered by an external power source and actively sends signals to the RFID reader. Active RFID mainly works in higher frequency bands such as 900 MHz, 2.45 GHz, or 5.8 GHz. The long-range and high-efficiency of active RFID makes it essential in some applications that require high-performance and large-scale properties.

Since the human body reflects the RFID signals, many studies apply RFID in human activity sensing [10,11,12,13]. For example, FEMO [14] calculates phase difference by measuring the phase of the reflected signals of the tags mounted on the dumbbells and establishes the relationship between the fitness actions and the phase differences. The pros and cons of using RFID in human activity detection and recognition are summarized as follows: 

Pros: RFID uses the principle of electromagnetic induction, so its wireless sensing ability is less affected by the environment. It can be used even in harsh conditions.

Cons: Many researches value the low-cost features of tags. However, RFID solutions are only working with the assistance of expensive readers.

### 2.2. FMCW

Frequency modulated continuous wave (FMCW) performs continuous modulation on the frequency of the transmitted signals. According to the pattern of triangular waves, the distance of the object can be estimated by leveraging the time difference and frequency difference between the transmitted and received signals. The signal frequency difference is relatively low, generally kHz, so the processing hardware is relatively simple and suitable for data acquisition and digital signal processing. FMCW signals are widely used in human sensing [15,16,17]. For example, RF-Capture [15] uses a combination of FMCW and antenna arrays to estimate the distance and direction between humans and antennas to track human motions. The pros and cons of using FMCW in human activity detection and recognition are summarized as follows:

Pros:

1. High sensitivity: Phases are extremely sensitive to small changes in the object position, which help estimate the tiny vibration frequency of the target (e.g., vibrations of breathing and heartbeat).
2. High resolution: The wireless bandwidth determines distance resolution. FMCW radar usually has a large bandwidth, so it achieves a high distance resolution.

Cons: The range of measurement is relatively short, and it is difficult for the signal isolation of sending and receiving.

### 2.3. Wi-Fi

Wi-Fi infrastructures have been widely deployed nowadays. Therefore, Wi-Fi has become a hot research direction in the field of human activity sensing. At present, there are two main metrics used in Wi-Fi sensing. One is received signal strength (RSS), the other is channel state information (CSI). 

RSS represents the strength of the received signal. In general, the RSS value is inversely proportional to the signal propagation distance. As the propagation distance increases, the signal attenuation becomes more significant, resulting in a decrease in the RSS value measured by the receiver. At present, most commercial Wi-Fi devices support obtaining RSS from the MAC layer, which measures the quality of the channel link. Sigg et al. [18] adopt RSS to recognize four activities, including lying down, crawling, standing, and walking with an accuracy of 80%. WiGest [19] leverages RSS values to sense in-air hand gestures and achieve a recognition accuracy of 96%.

Channel state information (CSI) launches from the IEEE 802.11n standard. Its core technologies include multiple-input multiple-output (MIMO) and orthogonal frequency-division multiplexing (OFDM). After dividing the limited spectrum resources into subcarriers, space-time diversity technology is applied to reduce the noise interference of the signal in space, and the communication capacity increases when using multiple antenna pairs. CSI represents the frequency response of each subcarrier of every antenna pair. CSI requires equipping with particular types of wireless network cards (e.g., Intel 5300 or Atheros Ath9k).

**Figure 2:**
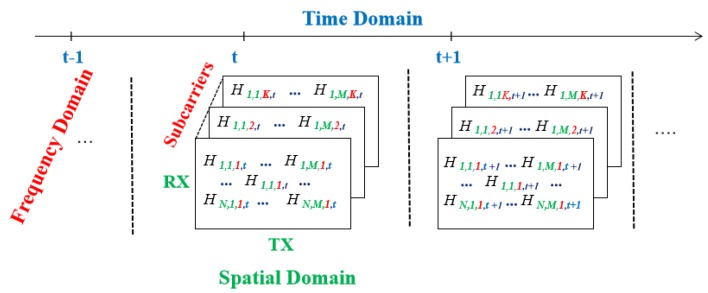
Four-dimensional CSI matrix of MIMO-OFDM channels.

In Figure 2, *H* represents the CSI information in MIMO-OFDM channels, which is a four-dimensional volume. *N* represents the antenna number at the transmitter, and *M* is the antenna number at the receiver. The first two dimensions marked in green represent the spatial domain. The third dimension marked in red belongs to the frequency domain, which represents the number of subcarriers under each antenna pair. The number of subcarriers obtained with the Atheros CSI Tool is 56 and is 30 subcarriers with Intel CSI Tool. The last dimension in blue indicates the time domain. Table 3 shows a comparison between RSS and CSI.

Due to the fine-grained information provided by Wi-Fi CSI, the use of Wi-Fi motion sensing has become a hot topic in recent years [19,20,21,22]. This paper reviews a variety of models on the relationship between human action and Wi-Fi, including the Doppler model, Fresnel zone model, and raw signals model in Section 3. The pros and cons of using Wi-Fi in human activity detection and recognition are summarized as follows:

Pros: Wi-Fi infrastructures have been widely deployed. Due to the fine-grained information provided by CSI, Wi-Fi can sense tiny movements such as finger gestures.

Cons: Wi-Fi cannot support motion sensing and communication at the same time. Moreover, Wi-Fi is relatively less robust to the environment changes than other signals.

### 2.4. Visible Light Communication

With the increasing applications of visible light communication (VLC), several studies conduct human tracking with visible light [23,24,25]. Any opaque object will shadow the beam emitted by the light source, so the key idea of human sensing by VLC is to analyze the shadows cast continuously on the floor to infer the user’s posture. The pros and cons of VLC-based human sensing are as follows.

Pros:

1. Low cost: VLC uses low-cost, high-efficiency photodiodes (LED), which can reuse the existing lighting infrastructure.
2. High transmission efficiency: VLC transmission process is fast and not subject to electromagnetic interference.

Cons:

1. Deployment effort: For perception accuracy, it needs to deploy hundreds of photodiodes.
2. High maintenance costs: Photodiodes age fast and have a weak anti-fouling ability. In order to ensure the perception accuracy of human actions, it is necessary to replace the old photodiodes in time, resulting in higher maintenance costs.
3. Vulnerable to ambient light: different intensity levels of ambient light may push photodiodes up into the saturation region, affecting the accuracy of motion perception.

### 2.5. Acoustic

The speakers and microphones of commercial off-the-shelf smart devices can generate and receive continuous sound waves. The human motion may affect the propagation of sound waves and create the phase difference or Doppler shift on received sound waves. By analyzing the received sound waves, the researchers may analyze the movement distance or corresponding direction of the human motion, which makes it possible for acoustic-based human sensing [26,27]. The pros and cons of acoustic signals for human sensing are as follows.

Pros: Due to the lower propagation speed comparing to RF signals, acoustic sensing can achieve millimeter-level accuracy.

Cons: Acoustic signals are vulnerable to interference from other signals in the band, so the choice of user scenarios is harsh. The noise in the environment will affect the accuracy of motion estimation.

### 2.6. LoRa

LoRa is a radio frequency transmission technique based on a spread spectrum modulation derived from chirp spread spectrum technology, which enables long-range transmissions with low power consumption. LoRa offers a long communication range for up to several kilometers, with the ability to decode signals as weak as −148dBm [28]. Because the human body may affect the propagation of LoRa signals, LoRa signals can help realize human sensing [29,30,31]. The pros and cons of LoRa signals for human sensing are as follows.

Pros: LoRa has a high penetration capacity and a wide range of communication. The transmission distance of LoRa signals extends to about 3–5 times compared to the traditional radio communication distance so that it can be applied to the perception and detection of a wide range of targets.

Cons: The longer sensing range implies that the interference range is also longer due to the higher signal receiving sensitivity. The received signal is more complex to extract human motion because of the interference of many unrelated objects during sensing.

### 2.7. LTE 

LTE signals have almost seamless coverage everywhere, which can be used in wireless induction as an easy-to-receive signal source. The movement of the human body may cause a change in the CSI of the LTE signals so that LTE signals can help in human sensing [32,33]. LTE is also suitable for the fingerprint algorithm to realize human localization [34,35]. The pros and cons of LTE for human sensing and localization are as follows.

Pros: The base stations to transmit LTE are widely distributed, so LTE signals are easy to receive, both indoor and outdoor. LTE signal reception is stable and not easily disturbed by other signals so that it will be a stable and reliable signal for human sensing.

Cons: The distribution of LTE signal base station is far away, and the signal propagation has long delays and offsets, so the accuracy of human localization based on LTE is still questionable. Besides, LTE transmission contains other unrelated information, so LTE-based motion-sensing requires specialized algorithms to reduce noise and separate signals.

## 3. Modeling Human Activity with Wireless Signal

In order to apply wireless signals to sense a variety of human activities, the most critical issue is to understand the relationship between human behaviors and wireless signals, i.e., it is the first question of how human motion affects the propagation of wireless signals.

**Figure 3:**
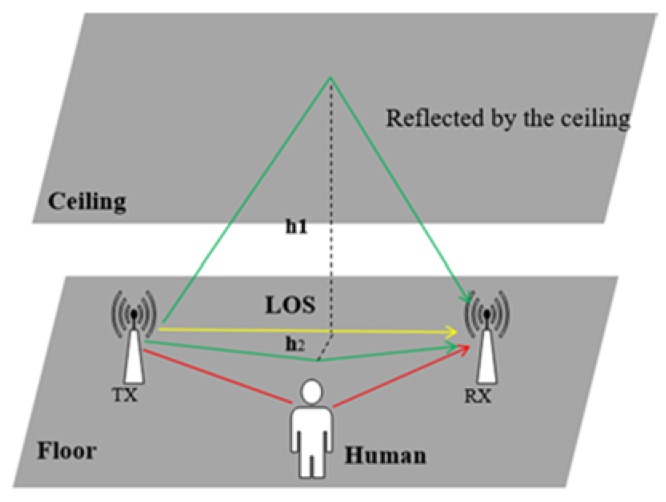
Indoor multi-path effect model.

In a typical indoor environment, shown in Figure 3, the yellow line represents the line-of-sight path (LoS) from the transmitter to the receiver, where the signal propagates directly without obstructions. In general, the signal attenuation on the LoS path is relatively small, so it’s received power is almost the highest among the multi-path propagations. The green line labels the paths of the signals reflected by the ceiling, the floor, or other obstacles inside the room. The red line shows the signals reflected by human motion, which is the focus of human motion sensing research. Signals from different paths experience with various delay, attenuation, or frequency shift, resulting in total signal distortion. These signals propagate along different paths and superimpose as a composite value at the receiver, called the multi-path effect [36].

In the time domain, the received signal Y(t) is a convolution of the transmitted signals X(t) and the channel’s impulse response (CIR) H(t), shown in Equation (1). H(t) is defined in Equation (2).$\;a_{n}\left(t\right)$ represents the amplitude attenuation of the *n^th^* path, $\tau_{n}\left(t\right)$ represents the propagation time delay,$\;e^{-j2\pi f\tau_{n}\left(t\right)}$ represents the phase offset on the *n^th^* path, and δ() represents a pulse function, respectively.

(1) Y(t)=H(t)⊗X(t)

(2) $$H\left(t\right)=\underset{n}{\sum }a_{n}\left(t\right)e^{-j2\pi f\tau_{n}\left(t\right)}\delta \left(t-\tau_{n}\left(t\right)\right)$$

Channel frequency response (CFR) is the frequency domain form of CIR, which represents the distortion that occurs on the frequency domain of the wireless signals. CFR can be obtained by performing Fourier Transformation (FFT) on CIR, as shown in Equation (3). Accordingly, in the frequency domain, the received signal spectrum Y(f) is the product of the transmitted signal spectrum X(f) and H(f), shown in Equation (4).

(3) $$H\left(f\right)=FFT\left[H\left(t\right)\right]=\underset{n}{\sum }a_{n}\left(t\right)e^{-j2\pi f\tau_{n}\left(t\right)}$$

(4) Y(f)=H(f)*X(f)

Among the variables involved in the above formula, X(t), X(f), Y(t), and Y(f) are measurable, while H(t) or H(f) are unknown. Hence, the motion recognition research seeks to derive H(f) and further deciphers the human action inside H(f).

There are three critical parameters inside Equation (3), which are the amplitude $a_{n}$, phase $\tau_{n}$, and frequency f. These three parameters are the primary aspects of wireless signals. For human motions, there are also three critical metrics: velocity, direction, and distance to the LoS path or the receiver (*RX*). Motion detection determines whether human motion exists, which often relies on coarse speed estimation in signals to find the speed change caused by human movement. Action recognition needs to identify the difference between multiple types of actions, so it needs more fine-grained speed information, distance range, and direction that an action span. Motion tracking needs to locate the position, direction, and distance to the receiver. Thus, this paper reviews the relationship model between phase, frequency, and amplitude of wireless signals and speed, direction, and distance of human activity. Table 4 provides a summary of the models between human activity characteristics and critical parameters of wireless signal propagation.

### 3.1. Phase

**Figure 4:**
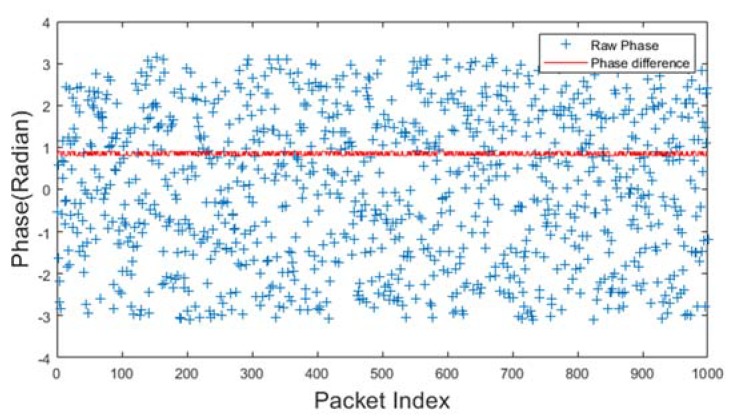
Phase difference distribution.

As human actions may change the length of some signal propagation path, resulting in phase offset, the phase information is used to deduct human motion. Figure 4 shows a raw phase sequence of the received signals with an Intel 5300 wireless network card when there is no human motion around. The raw phases are marked in blue in Figure 4. The raw phases from a single antenna are randomly distributed because it suffers from random phase offsets. The random offsets come from the immeasurable nature of carrier frequency offset (CFO) and sampling frequency offset (SFO) between transmitter and receiver. Therefore, the phase information cannot be used directly. The red dot represents the phase difference between two antennas at the receiver. It keeps stable without human existence. Previous research [37,117] show that the RF oscillator is frequency locked on a single commercial wireless NIC at startup. Therefore, there is no sampling frequency difference among the antennas on the same NIC. Thus, many researchers adopt phase differences as input for models associated with human motion and wireless signals [37,38,70,71,72]. The phase difference can derive the rough speed of the human movement.

Phase difference vs. human velocity: Higher the velocity, more intense the fluctuation of the phase difference. However, the phase difference can only roughly estimate human actions whose velocity varies significantly. The phase difference can be used to separated walking and running from other static actions, such as sitting, lying, and standing. For the action recognition problem, with the assistance of feature extraction, phase difference helps in distinguishing multiple actions [39,70,71,72]. Phase differences can hardly solve motion tracking problems because the spatial details (distance, direction) are hard to derive from phase differences.

### 3.2. Frequency

The human movement causes a change in the length of the reflection path, resulting in frequency shifts. By measuring signal frequency, the direction, speed, and distance involved with human movement can be deduced.

Frequency vs. human velocity: The relationship between human speed and signal frequency can be derived using the Doppler effect model. The Doppler effect indicates that when the human body moves relative to the transceiver, it produces a high frequency when approaching, or a low frequency when away from transceiver [55]. When the transmitter transmits the signal with the frequency $f_{t}$, the received signal frequency $f_{r}$ is calculated as Equation (5).

(5) $$f_{r}=f_{t}+f_{Doppler}$$

$f_{Doppler}$ is the Doppler frequency shift caused by human movement, which can be deduced because $f_{t}$ and $f_{r}$ can be measurable [56]. $f_{Doppler}$ models the human speed as Equation (6).

(6) $$f_{Doppler}=f_{t}\frac{v_{path}}{c}$$

**Figure 5:**
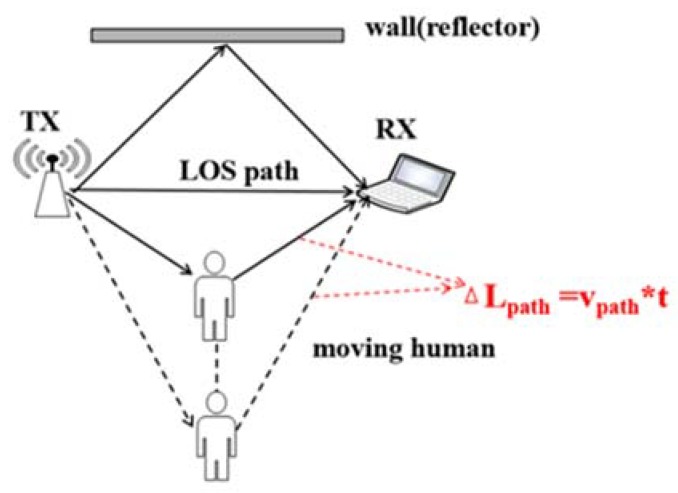
Path length change due to the human movement.

$v_{path}$ is the speed at which the length of the reflective path changes due to human movement, shown in Figure 5, called Doppler velocity. $v_{path}$ is the fundamental reason for the Doppler shift caused by human motion.

**Figure 6:**
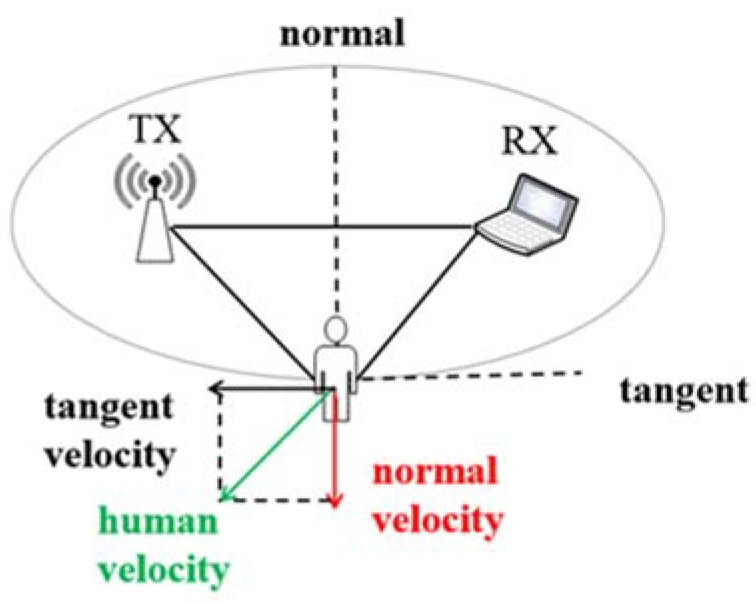
Geometrical relationship between human velocity and Doppler velocity.

We further explore the relationship between $v_{path}$ and human speed, shown in Figure 6. Figure 6 shows the deployment scenario that the human is on an ellipse with the transceivers on the focuses. From the ellipse property, the length of the reflected path by the human body is constant if the human moves along the ellipse. The speed of human movement can be divided into the tangential and normal speed. Tangential velocity directs along the ellipse, which does not change the length of the propagation path in a short period. Therefore, it does not generate a Doppler shift. On the contrary, normal velocity governs the human body away from the ellipse and thus produces a non-zero Doppler frequency shift.

**Figure 7:**
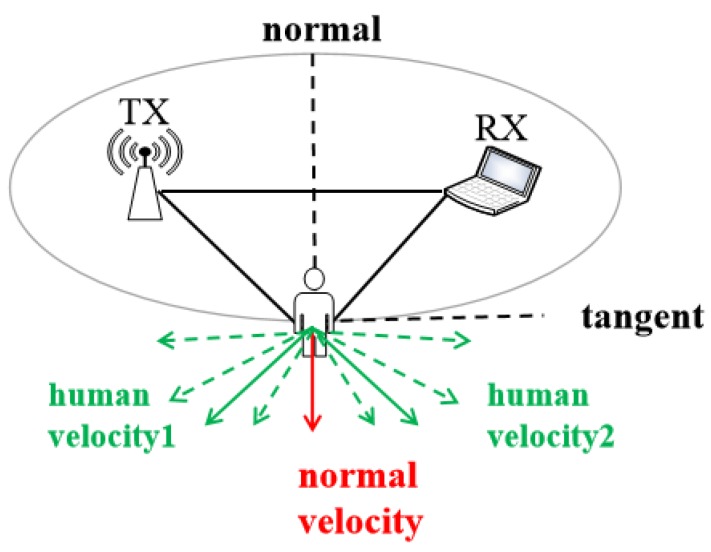
Directional ambiguity on symmetric velocity.

Frequency vs. human direction: In a fixed place, when people are moving at the same velocity in different directions, introducing distinct Doppler shifts, shown in Figure 7. $v_{normal}$ is calculated as Equation (7). Here θ indicates the direction of human movement.

(7) $$v_{normal}=v_{human}\;cos\theta$$

**Figure 8:**
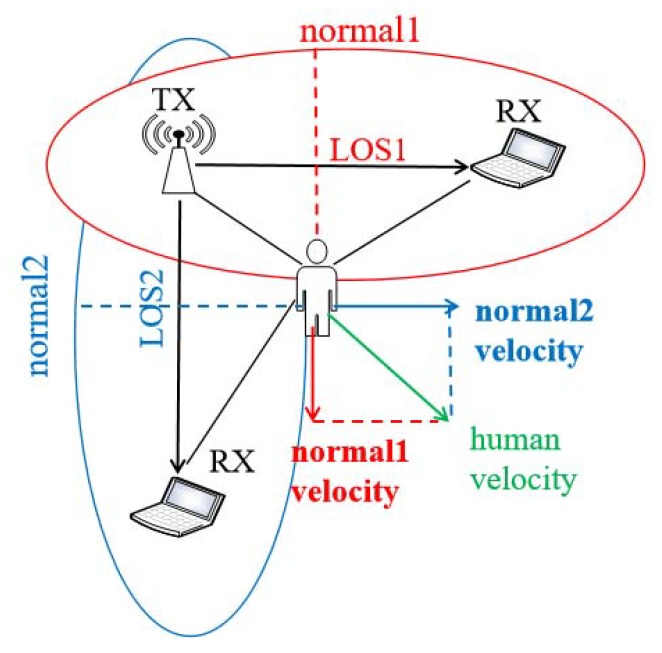
Doppler effect on multiple directions.

It is not possible to derive tangential velocity using only one pair of transceivers. WiDance [57] proposes a scheme to solve the tangential ambiguities by adding an orthogonal receiver, shown in Figure 8. When a person moves to the region Normal2, the length of the reflection path of the ellipse 2 becomes shorter, thereby generating a negative Doppler frequency shift. Similarly, when moving away from the region Normal2, the length of the reflection path to the left receiver becomes longer, causing a positive Doppler shift. A pair of transceivers can only judge the direction with the constant speed. In contrast, two orthogonal receivers can identify the direction of human movement at various speeds. For human orientation recognition, two orthogonal receivers can judge eight basic directions [57]. For the tracking of human movements, it requires more receivers to get more accurate spatial information about the direction of human motions [58].

Frequency vs. human velocity and distance: Accurately extracting a phase from an analytical signal requires that the signal contains only one frequency component at any given time. The chirp signal is an example of this type [106]. Human activities can be captured by radio signals reflected from the human body, which results in estimating the delay of the wireless signal from the transmitter to the reflected human body and back to the receiver. FMCW chirp has the advantage as a model that can compare different carrier frequencies at the same time. FMCW chirp converts the problem of estimating the time of flight (ToF) into a measurable frequency difference to capture human motion, which derives distance and speed information about human activities. For stationary people, only the distance needs to be measured. For the human motion, FMCW can measure the speed at which people move and their distances to the receiver.

1. Distance measurement when a person is still

**Figure 9:**
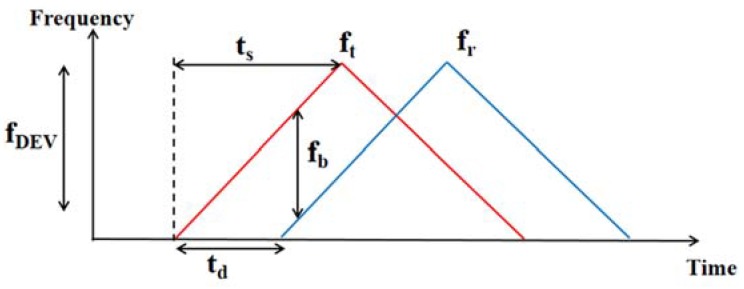
Principle of FMCW chirp

Figure 9 shows the principle of FMCW chirp, where the transceiver sends out signals repeatedly swept across a specific bandwidth. Here the transmitted and received waves are labeled in red and blue, respectively. Figure 9 describes the FMCW triangular wave produced by the frequency synthesizer. $f_{t}$ and $f_{r}$ represent the transmitted and received signal frequency, respectively. After signal reflection from the human body, the frequency difference $f_{b}$ is introduced as Equation (9). In Figure 9, the time difference $t_{d}$ can be measured, which are linear related to the distance between the human body and the transceiver $d_{RX}$. Label$\;f_{DEV}$ as the frequency sweep bandwidth and $t_{s}$ as the half cycle of the wave generated. According to the triangle similarity of Equation (10), $d_{RX}$ is calculated as Equation (11).

(8) $$f_{b}=f_{t}-f_{r}$$

(9) $$t_{d}=\frac{2d_{RX}}{c}$$

(10) $$\frac{f_{b}}{t_{d}}=\frac{f_{DEV}}{t_{s}}$$

(11) $$d_{RX}=\frac{f_{b}\cdot c\cdot t_{s}}{2\cdot f_{DEV}}$$

2. Speed and distance measurement

**Figure 10:**
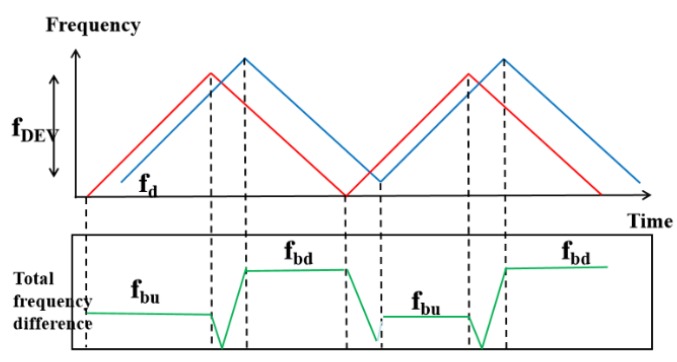
Frequency deviation on FMCW chirp on human motion.

In the scene of human movement, it needs to take Doppler shifts into account. In Figure 10, the frequency difference can be calculated as $f_{r}\;-\;f_{t\;}$, which changes regularly over time. Here $f_{bu}$ and $f_{bd}$ represent the frequency difference of the stabilization phase as Equations (12) and (13). Combining the Doppler model of Equation (14), the distance *d_RX_* and the speed of human movement *v_human_* can be calculated as Equations (15) and (16). FMCW chirp is usually used in conjunction with the antenna array to solve human tracking problems (more details in Section 3.3).

(12) $$f_{bu}=f_{b}-f_{Doppler}$$

(13) $$f_{bd}=f_{b}+f_{Doppler}$$

(14) $$f_{Doppler}=\frac{2\cdot f_{t}\cdot v_{human}}{c}$$

(15) $$d_{RX}=\frac{c\cdot t_{s}}{2\cdot f_{DEV}}\left(f_{bu}+f_{bd}\right)$$

(16) $$v_{human}=\frac{c}{4f_{t}}\left(f_{bd}-f_{bu}\right)$$

### 3.3. Amplitude

As the presence of human motion changes the pattern of multi-path propagation, the amplitude attenuations of the signals on different propagation paths are varying. This section presents the relationship between human motion and signal amplitude.

Amplitude sequence is accessible on commercial Wi-Fi devices without special equipment required. The amplitude value measured by the receiver is the superposition of the received signals from all the propagated paths, which is a more intuitive index compared to phase and frequency. WiFall [73] finds that the impact of human activity on different amplitude streams varies over time. Furthermore, the amplitudes of subcarriers among adjacent frequencies share more similarities than those with larger frequency gaps. The amplitude characteristics of signals can deduce distance direction, and speed of human motion.

**Figure 11:**
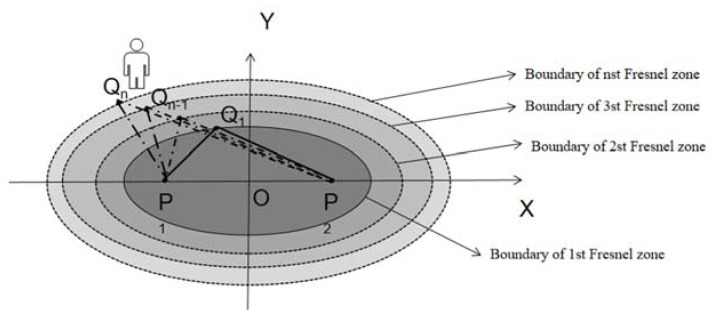
Fresnel zone model.

Amplitude vs. human distance: The Fresnel zone model can be used to deduce the relationship between amplitude and the distance of human motion. Fresnel zone is a series of concentric ellipsoidal with two foci corresponding to the transmitter and receiver antennas, shown in Figure 11. $P_{1}$ and $P_{2}$ are the locations where two radio transceivers reside. $Q_{n}$ is a point on the *n^th^* ellipse. For a given radio wavelength *λ*, the boundary of the *n^th^* Fresnel zone is calculated as Equation (17).

(17) $$\left|P_{1}Q_{n}\right|+\left|P_{2}Q_{n}\right|-\left|P_{1}P_{2}\right|=\frac{1}{2}n\lambda$$

**Figure 12:**
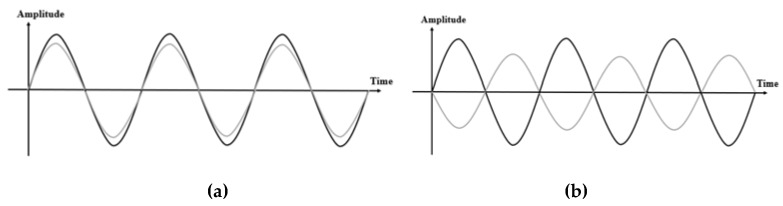
Signal amplitude with the human residing on the boundaries of Fresnel zones of odd and even numbers (**a**) odd zone (**b**) even zone.

Zhang et al. [107] show that when the length difference between the two paths is of one wavelength *λ*, the phase difference between them is *2*π. The received signals can be viewed as the superimposition of two major components, including one from the LoS path $P_{1}P_{2}$ and the other from the reflected path $P_{1}Q_{n}+P_{2}Q_{n}$. It is worth noting that for reflected signals, the phase reverses by π. The total phase difference equals to the sum of the phase difference between LoS and the reflection path and the phase difference π caused by reflection. Therefore, when a person resides in the odd-Fresnel zone boundary, the overall phase difference is 2nπ, which will reinforce the strength in the received signals, shown in Figure 12a. On the contrary, when a person appears in the even-numbered Fresnel zone boundary, the total phase difference is *(*2n+1*)*π, which weakens the composite signal amplitude, shown in Figure 12b. 

As n increases, the magnitude of the peaks and valleys decreases. Thus, when a person crosses the boundaries of several Fresnel zones in turn, there will be the peaks on signals when the phase difference is 2 π, 4 π, … 2nπ and the valleys when the phase difference is 3 π, 5 π, … (2n+1)π. By observing the peaks and valleys of the amplitude series, it is possible to determine whether a person locates in an odd or even Fresnel zone boundaries and the coarse distance from the LoS path [108]. For subtle human movements (breathing or finger gestures), the movement distance is often small so that the corresponding amplitude changes are difficult to distinguish. Niu et al. [118] propose adding multiple virtual paths to make a weak amplitude change more drastic and easy to judge.

Amplitude vs. human direction: The calculation of human direction from amplitude measurements have two models as follows. 

1. Fresnel zone model

**Figure 13:**
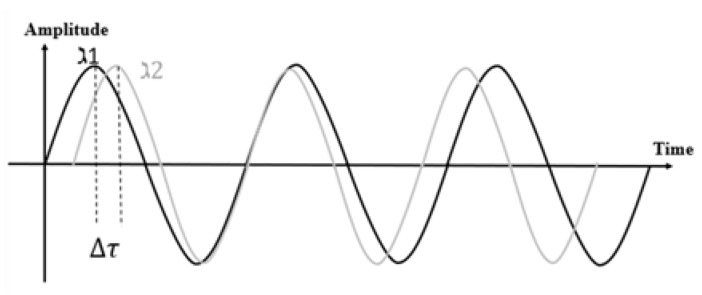
Delayed waveforms for subcarrier 1 and 2.

A single-frequency carrier cannot deduce the direction of human motion. With Wi-Fi MIMO-OFDM technology, multi-subcarrier can help calculate the direction of human action. Each subcarrier will create its Fresnel region independently. These multi-frequency Fresnel zones share the same foci and shape but different sizes. The subcarrier with a shorter wavelength has a smaller ellipsoid than the neighbor subcarriers. Therefore, the peaks and valleys of different subcarrier waveforms appear at different times, causing their waveforms to have phase differences. Figure 13 shows the example of the signal waveforms received on two adjacent subcarriers when a person crosses the Fresnel zone inward. By adopting cross-covariance in the sliding window to calculate the time delay between the waveforms of subcarriers, the positive delay means walking inwards and vice versa.

2. Antenna array

**Figure 14:**
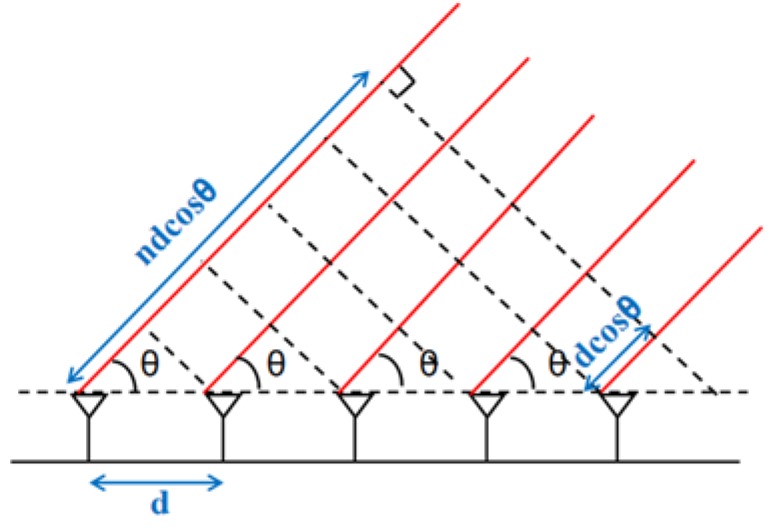
AoA model with the antenna array

The antenna array can measure the amplitude on multiple antennas to obtain the angle of arrival (AoA) of the signal, thereby deriving the direction of human motion. In Figure 14, when the signals arrive at a certain angle, there is a difference in the path length for the signals received by the different antennas. Variations in path length result in various delays *τ*, so there is a phase difference among the signals for different antennas. By measuring the received signals on every antenna, the power at any given angle *θ* can be obtained through Equation (18).

(18) $$P\left(\theta \right)=\left|\overset{N}{\underset{n=1}{\sum }}y_{n}e^{j2\pi \frac{nd\;cos\theta }{\lambda }}\right|$$

d is the distance between two antennas, λ is the signal wavelength, and ndcosθ indicates the signal propagation path length of the *n^th^* antenna. The arrival angle θ of the received signal can be derived when *P*(θ) maximizes, which reflects human motion direction. When combining with the speed model of FMCW chirp, human motion tracking can be tackled. ArrayTrack [119] deduces the relationship between the length of the signal propagation path and the phase change.

3. Amplitude vs. Human Velocity

CARM [36] proposes the CSI-Speed model, which correlates amplitude with motion velocity. Depending on whether the wireless signal is affected by human movement, the channel frequency response can be divided into static parts and dynamic parts. The static parts $H_{s}\left(f,t\right)$ is not affected by human actions, so it keeps a constant value. The dynamic part $H_{d}\left(f,t\right)\;$is time-varying because the emergence of human actions will change multiple signal propagation paths. $H_{d}\left(f,t\right)$ is defined as Equation (19).

(19) $$H_{d}\left(f,t\right)=\underset{k\in P_{d}}{\sum }a_{k}\left(f,t\right)e^{-j2\pi f\tau_{k}\left(t\right)}$$

$a_{k}\left(f,t\right)$ represents a compound value of amplitude attenuation and initial phase offset of a signal passing through the *k^th^* path. $\tau_{k}\left(t\right)$ is the phase difference caused by the propagation delay of the *k^th^* path.

When a person moves a small distance from time 0 to t, resulting in the length of the *k^th^* path changes from $d_{k}\left(0\right)$ to $d_{k}\left(t\right)$. $\tau_{k}\left(t\right)$ can be defined as $\tau_{k}\left(t\right)=\frac{d_{k}\left(t\right)}{c}$. As $\lambda =\frac{c}{f}$, $e^{-j2\pi f\tau_{k}\left(t\right)}$ can be written as $e^{-j2\pi \frac{d_{k}\left(t\right)}{\lambda }}$. When the path length changes one wavelength, the phase shift of the received signal is 2π.

The complex value H(f,t) at the receiver can be calculated as Equation (20). $e^{-j2\pi \Delta ft}$ indicates the phase difference caused by the carrier frequency shift Δf between transmitter and receiver. When a person moves at a constant velocity in a short period, the rate of change in path length is also constant $v_{k}$. The instantaneous CFR power is derived as Equation (21). Here $\frac{2\pi d_{k}\left(0\right)}{\lambda }+\phi_{sk}$ and $\frac{2\pi \left(d_{k}\left(0\right)-d_{l}\left(0\right)\right)}{\lambda }\;+\phi_{kl}$ are constant values, which represent initial phase offsets. The total CFR is the sum of a series of sinusoids and static constants, where the frequency of the sinusoids is a function of the speed $v_{k}$ in path length change caused by human movement. By measuring these frequencies and then multiplying their wavelengths, the human motion speed $v_{k}$ can be derived. Hence, the CSI-Speed model infers the human motion velocity from the measurable CFR.

(20) $$H\left(f,t\right)=e^{-j2\pi \Delta ft}\left(H_{s}\left(f,\;t\right)+H_{d}\left(f,t\right)\right)$$

(21) $$\begin{array}{ll} {\left|H\left(f,t\right)\right|}^{2}=\underset{k\in P_{d}}{\sum } & 2\left|H_{S}\left(f,t\right)a_{k}\left(f,t\right)\right|\cos\left(\frac{2\pi v_{k}t}{\lambda }+\frac{2\pi d_{k}\left(0\right)}{\lambda }+\phi_{sk}\right)\\ & +\underset{k\neq l,k,l\in P_{d}}{\sum }2\left|a_{k}\left(f,t\right)a_{l}\left(f,t\right)\right|\cos(\frac{2\pi \left(v_{k}-v_{l}\right)t}{\lambda }+\frac{2\pi \left(d_{k}\left(0\right)-d_{l}\left(0\right)\right)}{\lambda }\\ & +\phi_{kl})+\;\underset{k\in P_{d}}{\sum }{\left|a_{k}\left(f,t\right)\right|}^{2}+{\left|H_{S}\left(f,t\right)\right|}^{2} \end{array}$$

### 3.4. Raw Signals

Due to the multipath effect, the received signals are the superposition of the propagated signals along different paths. If each reflection path affected by human motion can be resolved from the received signals, it will definitely improve performance for passive human localization and motion tracking. mD-Track [116] constructs the path resolving model to jointly estimate the multi-dimensional parameters of each reflection path, including angle-of-arrival (AoA), angle-of-departure (AoD), time-of-flight (ToF), and Doppler shift. 

mD-Track models the received signal as the superposition of signals along *L* distinct paths as Equation (22).$\;s_{l}\left(t;\nu_{l}\right)$ indicates the signal along the $l^{th}$ path where $v_{l}=\left[\phi_{l},\varphi_{l},\tau_{l},\gamma_{l},\alpha_{l}\right]$. Here ϕ, φ, τ, γ, and α represents AoA, AoD, ToF, Doppler shift and complex attenuation respectively. The goal of mD-Track is to estimate the path parameters $V=\left[\nu_{1},\nu_{2},\ldots ,\nu_{L}\right]$ for all L paths in *Y(t)*.

(22) $$Y\left(t\right)=\overset{L}{\underset{l=1}{\sum }}s_{l}\left(t;\nu_{l}\right)+W\left(t\right)$$

(23) $${\left(\phi ,\varphi ,\tau ,\gamma \right)}_{est}=arg_{v}\;max\left|z\left(\phi ,\varphi ,\tau ,\gamma \right)\right|$$

(24) $$z\left(\phi ,\varphi ,\tau ,\gamma \right)=\int_{T}e^{-j2\pi \gamma }F^{-1}\left\{g^{H}\left(\varphi \right)Hc{\left(\phi \right)}^{*}⊙LTF\right\}U^{*}\left(t-\tau \right)dt$$

mD-Track employs iterative parameter refinement for each propagation path in multiple rounds. During each round, the parameters of the current signal path are estimated through solving the optimization problem defined in Equation (23) and (24). Here *g(φ)* characterizes the phase relationship of the signal coming out of the transmitting antennas while the receive array steering vector *c(*ϕ*)* characterizes the phase relationship of the signal arriving at the receiving antennas. *H* is the CSI matrix, and *LTF* is the preamble according to the 802.11n standard [120]. *U(t)* is the residual signals after eliminating the estimated signals from the received signals. mD-Track points out that the proposed iterative optimization is a maximized expectation problem belonging to the EM family [121,122], which guarantees the converge. After separating each reflection path, the AoA, AoD, and ToF of each path can be used for human localization, while the Doppler shift of each path can be used in motion tracking.

## 4. Signal Preprocessing

This section presents the signal preprocessing methods for motion sensing with wireless signals in recent years, including noise reduction, calibration, and redundant removal. Table 5 provides a summary of the signal preprocessing techniques.

### 4.1. Noise Reduction

The raw signals extracted from the PHY layer are very noisy due to hardware defects or some particular noise in the environment. To use wireless signals for human motion sensing, eliminating as much noise as possible is the first step.

Time-domain filtering: Moving average filter and median filtering are simple methods for time-domain analysis. Each data point is replaced by an average or median value of adjacent data points. For example, SEARE [74] adopts a moving median filter to smooth the CSI waveform and eliminate outliers. The weighted moving average filter utilizes the scheme that the values closer to the processed point should occupy a higher weight. HuAc [75] smooths and reduces the serrates of CSI waveform by using the weighted moving average filter. Single-Sideband Gaussian (SSG) applies a convolution to smooth the raw signal, which is used to preprocess the CSI waveform in [76]. The Savitzky–Golay filter computes the local polynomial least square fitting in the time domain to filter out noise while ensuring that the shape and width of the signal are unchanged. Zhang et al. [108] use the Savitzky–Golay filter on CSI signals to fit a continuous subset of adjacent data points so that the CSI signal is denoised without distorting the signal waveform.

Some outliers may not be filtered and will affect the subsequent processing. Local outlier factor (LOF) is employed to find anomalous points by measuring the local density of the collected signals. For example, WiSome [59] uses LOF to find and remove outliers on CSI streams. The Hampel filter computes the median *mi* and standard deviation *σ_i_* of adjacent data points. If |*xi* − *mi* |/*σ_i_* is larger than a predefined threshold, the current point *xi* is viewed as an outlier and replaced with the median $m_{i}$. EI [77] uses the Hampel filter to remove outliers.

Frequency domain filtering: The frequency caused by human motion is usually much lower than the frequency of impulses and burst noises. In order to choose signals for a specific frequency band, some filters of frequency domain analysis are applied. Butterworth low-pass filters and Passband filters are widely used to remove high-frequency noises. WiChase [40] uses a Butterworth filter with a relative flat magnitude response on CSI signals so that the phase information can keep unchanged. Wavelet filter removes noises from the signals without losing high-frequency components, especially when activity details are required. WiSome [59] considers that the frequency of CSI signals is generally high, and the Doppler frequency shift caused by human movement is relatively low. It applies the Wavelet filter to extract the information from the low-frequency layer. Birge–Massart filter estimates the adaptive density of non-parameters after wavelet transformation to obtain a threshold. WiG [78] uses Birge–Massart filter to smooth the signal sequence and simultaneously capture the time and frequency domain information. Kalman filter can achieve the estimate of the motion signal, even the frequency band of motion and noise are somehow mixed. WiDeo [41] uses the Kalman filter to improve the localization accuracy during tracking.

### 4.2. Calibration

Due to the inconsistency among filtered signals, calibration is the second step of signal preprocessing.

Interpolation: The receivers may obtain non-uniform sequences due to weak signals through-wall or from non-LoS paths, which have packet loss and transmission delays. For a relatively stable sampling frequency, the received signal sequence often needs to be interpolated. RT-Fall [37] uses interpolation to eliminate the discontinuity in CSI values caused by the uneven arrival interval of data packets, thereby obtaining uniformly sampled sequences.

Normalization: The imbalance of signal distribution comes from the different ranges on the various dimension. Normalization unified the value scale by normalizing from 0 to 1 proportionally. Motion-Fi [79] uses the normalization of the raw signals before performing signal processing.

**Figure 15:**
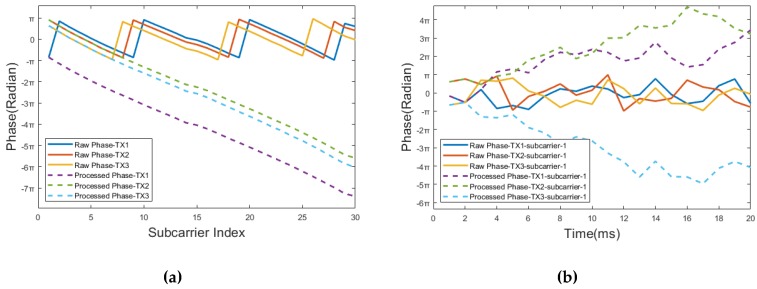
Raw vs. pre-processed CSI phase (**a**) CSI phase vs. subcarrier index (**b**) CSI phase vs. sampling time.

Phase calibration: The filtered phase is folded due to the nature of the inherent phase periodicity, which needs to transform the raw phase into the real value. Figure 15 shows the raw phases of CSI for the three antennas at the receiver. It shows that the raw phase sequences of the three antennas are folded, in the phase range [−π π]. SignFi [21] obtains the linear transformation of CSI phases from different subcarriers of different antennas by using a linearly unwinding method. Different phase patterns over time can be calculated by applying a linear unwinding method, shown in Figure 15. Besides, the absolute phases differ because each radio chain connects to different RF oscillators. Phaser [117] proposes a phase autocalibration algorithm that corrects the phase offsets between the different radio oscillators at an AP.

### 4.3. Redundant Removal

After the above pre-processing, the signal sequence still contains some redundant information that is not related to human activity. The removal of such unnecessary details will reduce computation complexity and sift out the signal segment tightly associated with human activities.

PCA-based subcarrier selection: The CSI measurements are highly correlated among subcarriers, and different subcarriers have different sensitivity for a given activity [42]. Thus, the most common method for redundant removal is to apply principal component analysis (PCA) on the CSI measurements to extract principal components. The principal components will capture the dominant variation caused by human activities.

Existing researches hold different views on principal component (PC) selection. Some solutions select the first PC, which contains the highest eigenvalue among all the PCs and may correspond to the features caused by human motions [42,58]. On the contrary, some researches choose to discard the first PC and preserve the second PC [36,80]. They assume that noises are primarily captured in the first PC. Moreover, some studies choose the third PC because it may have the highest motion-related signal to noise ratio [69]. Fang et al. [43] select the first two PCs because they empirically find these two PCs contain the majority of the total variance and thus preserve most information of the targeted activities. Melgarejo et al. [39] make use of the top-five PCs to provide distinguishable metrics.

Static environment partial removal: The static signal propagation paths are often treated as a constant in a short period, which is not affected by human activity. Thus, the static component inside the received signals is often removed by subtracting the constant value measured in the static environment without human actions [15,22,65].

Multipath mitigation: If the surrounding environment changes, such as moving a chair to another place or a person is moving around, the received signals will be different due to the various multipath effect for signal propagation, resulting in the signal pattern distortion for a given activity. WiFinger [20] finds that the signal reflection path usually has a longer propagation delay than the LoS path. Thus, to keep robust to the environment changes, WiFinger removes the signal components that have a longer delay with Inverse Fast Fourier Transform (IFFT).

## 5. Signal Segmentation

**Figure 16:**
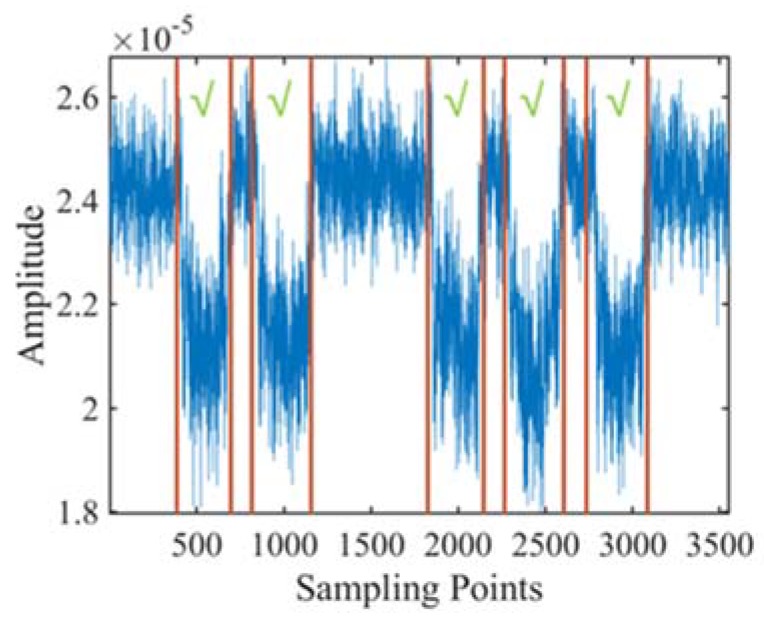
Segmentation example of the received amplitude sequence during five squats. Green check labels each squat segment. The red vertical lines label the start and end timestamp of each squat.

After pre-processing, the remained signals comprise motion segments and non-motion intervals. The non-motion interval inhibits from discovering the characteristics of signals affected by human movement. Figure 16 shows an example of the received signal sequence while a volunteer performs five squats. The start and end timestamps are labeled with red lines in Figure 16, which are captured by video analysis. Figure 16 shows that there is a long rest interval between the second and third squats, which may affect the accuracy of action detection and recognition. The segmentation target is to accurately find out the start and end timestamp for each human action inside the received signal sequence.

Precise segmentation for every single action from the signal sequence is the premise of accurate feature extraction and activity recognition. Because human action may induce high fluctuations in the received signals, the action segmentation is mainly based on thresholds. Hence, the action segmentation methods can be classified into two categories, time-domain based and frequency domain-based methods. Table 6 provides a summary of the signal segmentation methods.

### 5.1. Time-Domain Threshold

According to the metrics, time-domain thresholds use thresholds on phase difference, amplitude, statistic features, energy, and similarity comparison.

Phase difference threshold: Phase difference threshold implicitly makes use of the spatial information between the antenna pairs at the receiver [37]. MoSense [71] calculates the mean of the Euclidean distances among all subcarrier pairs using Equation (25).

(25) $$\bar{d}=\frac{\sum_{k=1}^{K-1}d_{k}}{K-1}$$

(26) $$T=\alpha \times \bar{d}$$

$d_{k}$ means the Euclidean distance of the *k^th^* and the *(k+1)^th^* subcarrier. The threshold T is chosen as Equation (26) where α is an empirical parameter.

In general, threshold cutting based on phase difference is used to cut walking and non-walking (such as sitting and standing) activities [81]. However, it is only suitable for signals propagation with high robustness to environmental changes.

Amplitude threshold: Amplitude thresholds are widely used in action segmentation with the advantage of low computation. WIAG [82] calculates the threshold through Equation (27).

(27) $$T=\left(\mu_{S}+\mu_{G}\right)∕2\;$$

$\mu_{S}$ means the average of absolute amplitudes of peaks when the person is stationary. $\mu_{G}$ is the average of absolute amplitudes of peaks when the person is moving. Amplitude thresholds can cut out the start and end of the simple human gestures (brush, phone, shake, push, pull, circle, etc.) or in-air finger gestures (circle left, right-left, up-down, infinity, open-close, etc.) contained in signal sequences [14,19,20,23,24,40,44,82,83,84,85,86,108].

In order to reduce the impact of environmental changes, the cutting modules extract features from the amplitude stream [44]. The threshold calculation on the amplitude difference is shown in Equation (28).

(28) $$\mu_{stable}^{i}+2\sigma_{stable}^{i}+3\varepsilon_{stable}^{i}\leq T^{i}$$

$\mu_{stable}^{i}$, $\sigma_{stable}^{i}$, and $\varepsilon_{stable}^{i}$ means the mean, standard deviation, and median absolute of *i^th^* gestures, respectively. Zhang et al. [108] set the threshold as the maximum variance when no activity exists to detect human motion. The amplitude variance threshold is generally used to cut the rest interval between fitness actions (push-up, sit-up, etc.).

Statistics threshold: For avoiding misjudgments affected by outliers, statistics thresholds are used in activity segmentation. The significant variation in one sliding window indicates the presence of human activity. The statistical thresholds include variance, variation coefficient, correlation, and outlier.

Because the in-place action is of less physical movement than walking, resulting in different variances [81]. Cumulative moving variance among K subcarriers is defined as Equation (29).

(29) $$\nu =\overset{K}{\underset{k=1}{\sum }}\nu \left(k\right)$$

ν(k) is the moving amplitude variance on *K* subcarriers for each sliding window. It is a robust feature to detect the state transition between actions, which means dynamic gestures lead to noticeable fluctuation in amplitude variance among sliding windows [81]. Cumulative moving variance threshold can generally cut out in-place actions and walking activities [76,81].

The coefficient of variation (CV) threshold relies on the fact that CV can balance the difference caused by the environmental changes, defined as Equation (30). σ is the standard deviation, and μ is the mean. The CV threshold can be used in breath monitoring and motion detection [45].

(30) $$CV=\frac{\sigma }{\mu }$$

Because the variance ratio is relatively stable to environmental conditions, Gong et al. [38] apply a threshold on the combination of short-term variance ratio (SVR) and long-term variance ratio (LVR). SVR is defined in Equation (31), which detects the transient state of abnormal situations, i.e., whether human actions occur. LVR is defined in Equation (32), which monitors continuous abnormal conditions. ΔLT and ΔT represent long and short intervals, respectively. The method based on the combination of SVR and LVR cuts the interval between human action through the empirical thresholds.

(31) $$SVR=\frac{1}{K}\overset{K}{\underset{i=1}{\sum }}\left|\frac{cv_{\Delta T}^{i}}{cv_{\Delta T-1}^{i}}\right|$$

(32) $$LVR=\frac{1}{K}\overset{K}{\underset{i=1}{\sum }}\left|\frac{cv_{\Delta T}^{i}}{cv_{\Delta LT}^{i}}\right|$$

The correlations between subcarriers are also used as thresholds to detect the motion segment. The eigenvectors among subcarriers change randomly in the absence of human movement. On the contrary, when human actions exist, nearby subcarriers become similar and correlated. Wang et al. [36] further calculate the variance on the selected principal component as a threshold to detect human motions with Equation (33).

(33) $$E\left\{h_{2}^{2}\right\}∕\delta_{q_{2}}$$

$E\left\{h_{2}^{2}\right\}$ and $\delta_{q_{2}}$ represent the variance and the mean derivation on the second principal component after applying PCA on the CSI streams of all subcarriers. Thresholds are then determined empirically. This kind of threshold can detect the start or end of activities, including walking steps, finger gestures, and activities (walking and running, squat, sit down) [36,46,87].

Local outlier factor (LOF) is defined as Equation (34). p is the current data point, and *o* is a point near the p. k(p) represents the set of k-nearest neighbors of p. lrd (p) is the local density of p. The high LOF indicates abnormal sequence caused by human movements. LOF threshold can cut out the start and end of human actions, finger gestures (right, left, push, pull), and fall state [73,76,78].

(34) $$LOF\left(p\right)=\frac{\frac{1}{k}\sum_{o\in k\left(p\right)}lrd\left(o\right)}{lrd\left(p\right)}$$

Similarity threshold: Fitness workouts usually consist of a group of periodic actions. In order to segment repetitive motions from the signal sequence, it is common to compare the similarity of signal sequences. Autocorrelation can be used to describe the degree of correlation. Guo et al. [88] adopt the autocorrelation to separate non-workout from a group of workout activities (biceps curl, leg stretch, leg press) through empirical thresholds. Meanwhile, Motion-Fi [79] exploits dynamic time wrapping (DTW) in the time domain to cut out action segments and update templates alternatively. Motion-Fi [79] optimizes the matching template and the cutting segments alternately, and finally sets the empirical threshold to cut the repetitive periodic fitness actions. These periodic actions include squat, push-ups, sit-ups, leg-raise, etc.

Energy threshold: The signal energy in the presence of human action is often larger than the one without human actions. The power in each action segment will first increase and then decrease due to human action. The energy threshold adopts the empirical mode decomposition (EMD) and the Hilbert Huang Transform (HHT) to calculates the ratio of real-time energy to the energy sum of each window. It identifies the start and end of the driver motions (include nod, yawn, bend over, and make a call) to check if the driver is fatigued [45].

### 5.2. Frequency-Domain Threshold

According to the Doppler model, there is a clear frequency shift when human motion appears. Hence, it is feasible to cut the action segment with the frequency threshold. Such methods need the assistance of time-frequency domain analysis, which can be further divided into three categories, including peak-based, energy-based, and spectrum-based.

Peak threshold: WiDance [57] computes the average sum of absolute Doppler frequency shifts of the two links and detects the prominent peaks. The user action causes a pair of peaks or valleys in Doppler frequency shifts with significantly different amplitudes. The two adjacent peaks are grouped as one complete action. This method can detect eight basic human movement directions (up, down, left, right, top left, top right, bottom right, bottom left). WIMU [69] uses STFT to analyze the frequency spectrum and counts the number of the frequency with magnitude larger than the threshold. An increase and decrease in the frequency number correspond to the beginning and end of the action, respectively. WIMU sets the threshold using the three-sigma rule as Equation (35). This cutting method is used for numeric gestures from one to six [69].

(35) T=μ+3σ

Energy threshold: Guo et al. [88] apply the power spectral density (PSD) in the frequency domain and calculate the normalized short-time energy (STE) to segment the signals. STE can be derived through Equation (36), where V (i) represents cumulative PSD, and W (n) is the windowing function. Each peak on STE represents a fitness repetition. These actions include biceps curl, leg stretch, leg press, etc.

(36) $$E_{sqr}=\overset{\infty }{\underset{i=-\infty }{\sum }}{\left[V\left(i\right)W\left(n-i\right)\right]}^{2}$$

Similarity threshold: WiFit [56] uses the Doppler displacement derived from the Doppler frequency shift for an impulse-based cutting. It uses DTW to calculate the similarity of each impulse. The impulse which meets the similarity threshold is considered as a repetitive action. This method can cut out three fitness exercises, including squats, sit-ups, and push-ups.

Kullback–Leibler (KL) divergence leverages the fact that the distribution of amplitudes within each window should be similar when there are no human actions. Conversely, the amplitudes change rapidly and show a completely different distribution with human motions. KL divergence is defined in Equation (37), which represents the loss of information when fitting the real probability distribution *P* using the theoretical distribution *Q*. KL divergence threshold can cut out fitness activities and rest intervals. These fitness exercises contain concentration standing bicep curl, seated triceps press, and flat bench bicep curl, which includes a unique arm pattern in each action [10].

(37) $$D_{kL}\left(P\|Q\right)=\underset{i}{\sum }P\left(i\right)\cdot ln\frac{P\left(i\right)}{Q\left(i\right)}$$

## 6. Feature Extraction

Feature extraction is the core step in motion recognition, which directly affects the recognition robustness and accuracy. Because human action is often buried inside the received signals, as discussed in Section 3, it is necessary to extract the features representing the action from the signal sequence. The extracted features can be classified into the time domain, frequency domain, time-frequency domain, and spatial domain features, which are summarized and compared in Table 7.

Time-domain features: Most time-domain features directly apply statistics. Calculating time-domain features usually takes the input of amplitude, phase, or phase difference whose computation costs are small. The statistics feature often characterize the shape of the received waveform in the time domain. There are a large amount of studies [13,37,40,42,43,70,72,73,76,79,80,83,85,88,89,90,108] that extract time-domain statistical features (maximum, minimum, mean, standard deviation, kurtosis, skewness, variance, median and median absolute deviation, percentiles, root sum square, interquartile range) from amplitude or phase streams. In particular, WIG [78] extracts the above time-domain features from anomaly data series obtained by the LOF-based segmentation method. RT-Fall [37] adds two extra features of the time lag and power decline ratio to the basic statistical features in phase difference streams. These above features are usually used by machine learning classifiers (SVM, HMM, random forest, etc.). Besides, many studies [10,19,20,39,55,74,81,89,91] with template matching classification often use the entire time-domain waveform (amplitude-waveform, phase-waveform, etc.) as a feature. For example, SEARE [74] uses the amplitude-waveform in the template matching.

Frequency-domain features: The frequency domain analysis may extract the signal characteristics from a deeper level than the time domain. Compared to time-domain methods, frequency-domain analysis usually requires a large amount of computation. Typically, the signal is transformed in the frequency domain, and then some useful parameters are extracted as the features to the frequency domain. Frequency domain features describe the magnitudes of various frequency components contained in the mixed signal.

HeadScan [43] extracts dominant frequency as the features. FallDeFi [92] proposes a frequency-domain feature called fractal dimension, which is robust to environment changes. Zeng et al. [85] calculate the frequency domain energy as a feature. Humantenna [83] and Sekine et al. [12] apply all the low FFT coefficients as features. WiFit [56] puts forward two useful features—Doppler velocity intensity and normalized Doppler velocity range. WiSee [55] and FEMO [10] use the Doppler shifts in the classification of template matching. Many studies [43,70,72,73,80,90,92] extract spectral entropy from frequency streams as the classification features.

Time-frequency domain features: Time-frequency domain analysis describes the proportion of specific frequency components that the signals contain at different times. Discrete wavelet transformation (DWT) is a representative of time-frequency domain analysis. DWT has good trade-off on time and frequency resolution, so both high-speed and low-speed motion can be captured. WiMotion [42] performs DWT on the amplitude sequence based on the first-order Daubechies wavelet. It takes the approximation coefficients of the third layer as the feature. WIAG [82] uses Daubechies wavelet on PCA components to extract three layers of detail coefficients as features. WiHear [93] firstly uses a four-order Symlet wavelet, then apply a feature selection scheme to extract the most representative features from wavelets. CARM [36] utilizes DWT to decompose the PCA components into 12 levels that span the frequency range from 0.15Hz to 300Hz. CARM extracts the energy of each layer as the features to imply the speed of path length changes caused by human movement. WiGest [19] extracts the spectrogram pattern for the template matching method. DELAR [47] proposes a deep learning framework of image processing on spectrograms to classify actions.

Another analysis method in the time-frequency domain is a combination of empirical mode decomposition (EMD) and Hilbert–Huang Transform (HHT). EMD is a self-adaptive signal processing method that decomposes data into intrinsic mode functions (IMF), which are symmetric concerning the local zero mean, and have the same numbers of zero crossings and extremums. Each IMF represents the type of oscillation pattern embedded in the signal. By applying HHT to each IMF, the instantaneous frequency can be acquired. Mohammed et al. [80] extract six features (mean, maximum, standard deviation, percentiles, median absolute deviation, and entropy) for both the amplitude and phase subsequence after HHT decomposition. WiFind [45] extracts eight features, including max total frequency energy, mean of total frequency energy, standard deviation of total frequency energy, median absolute deviation of total frequency energy, length of the breath patterns extracted from principal component, and mean, STD, MAD of the breath pattern to detect car driver fatigue.

Spatial domain features: For the application of human localization and tracking, it is essential to capture the spatial information such as the direction and distance of the human body at a certain moment. AoA and ToF are two typical spatial features. By exploiting the antenna array, AoA can be derived from the phase difference of the arriving signals between multiple antennas, refer to Section 3.3. Because the signal is approximately the speed of light, ToF is usually tiny and challenging to measure directly. FMCW chirp measures the frequency difference between two consecutive triangular waves, which help to estimate ToF, as discussed in Section 3.1. For localizing a human in three-dimensional space, WiTrack [66] leverages the T shape directional antenna array to estimate the AoA and ToF of FMCW signals. Chronos [132] achieves decimeter-level human localization with a single Wi-Fi AP to estimate ToFs from multiple frequency bands. WiDeo [41] calculates the ToF, amplitude, and AoA of the signals reflected from the human body by using the backscatter sensor.

## 7. Activity Classification

The features extracted from action segments will be further applied in classifier to recognize human activities. This section focuses on techniques for activity classification, including template matching, machine learning, and deep learning. In terms of training options, these classification methods can be divided into training-free, training-once, and multiple times of training [133]. Template matching recognition is often training-free [10,19,20,39,55,74,91,93,94]. Training-once classification requires the valid features robust to the variations in the surrounding environment [12,13,32,37,40,42,44,45,47,48,49,56,59,60,70,72,73,75,76,78,79,83,84,89,90,92,127,130]. Deep learning automatically extracts features, which often requires only one time of training [16,17,21,48,59,67,77,88,95,96,108]. For features that change dramatically with the environment, multiple times of training should be performed when there is some change in the environments. Wi-Multi [97] propose a three-phase system using CSI according to the size of available training samples. Table 8 describes a summary of the activity classification techniques.

### 7.1. Templated Matching

Since template matching is a real-time training-free method, its input should be sufficient pre-processed and segmented out signal sequence. The template matching method has to pre-store the templates, which are not suitable for a large number of templates. So template matching is more applicable for recognizing action with fewer categories and of short time series per template. Because human gestures have short durations, the template matching has been widely used in gesture recognition and simple motion recognition.

These methods calculate the distance between the action sequence and the known template and measure it based on the similarity thresholds. If the distance is less than the threshold, the action sequence is classified into some known type. According to whether the time series are of fixed length, these methods can be further divided into fixed and different length template matching methods.

Fixed length: The difference among fixed template matching is using different distance calculation. Euclidean distance is the simplest distance evaluation. For example, WiGest [19] firstly encode the rising edges to positive signs, falling edges to negative signs, then apply Euclidean distance for gesture matching. WiGest can recognize four gestures (right-left, up-down, infinity, open-close) performed by fingers.

Compared to European distance, Earth mover’s distance (EMD) can measure the similarity between two probability distributions. It calculates the minimal cost to transform one distribution into the other [70,81,89]. Thus, lower EMD means that the two distributions are more highly correlated, indicating that the activity to be identified belongs more likely to the template activity. For example, E-eyes [81] employs the EMD to quantify the similarity of testing CSI measurements and the known in-place activity profiles for in-place human daily activities (cooking, eating, washing dishes, studying, brushing, bathing, etc.) classification.

Jaccard coefficient between the two matrices is the ratio of the number of sample intersections and the number of sample syntheses, which compares similarities between limited sample sets. The higher the Jaccard coefficient value, the higher the sample similarity. WIMU [69] measures the Jaccard similarity coefficient of every two samples and calculate the average of all Jaccard coefficients to distinguish six digital finger gestures (from one to six).

Different length: The length of two signal sequences for the same action is often different due to the differences in duration, direction, and speed. The typical template matching method for different-length series is dynamic time wrapping (DTW) [10,20,39,42,55,74,91,93].

DTW solves the length problem by optimally calculate the distance between two series by stretching and alignment. For finger gesture classification, Mudra [91] classify nine finger gestures (shoot, pick, come, tap, double-pick, double-tap, circle, twist, go) through DTW. Melgarejo et al. [39] classify four-finger gestures (down, continue, browser, next) by DTW. WiFinger [20] achieves the eight finger gestures (zoom out, zoom in, circle left, circle right, etc.) recognition with DTW. Besides, WiMotion [42] classifies six daily human activities (bend, hand clap, walk, phone call, sit down, squat) with DTW. WiSee [55] realizes nine human gestures (push, dodge, strike, pull, drag, kick. etc.) recognition through DTW. For fitness activity classification, SEARE [74] recognizes four activities (dumbbell lift, squat, kick, boxing) through DTW. FEMO [10] applies DTW to 10 dumbbell exercises (concentration bicep curl, seated triceps press, flat bench bicep curl, etc.). In particular, WiHear [93] adopts DTW to achieve lip and tongue movement recognition.

### 7.2. Machine Learning

The classification based on machine learning needs signal preprocessing and feature extraction as its basis. Since the classification method of machine learning requires training the model, its time complexity is higher than that of template-based methods. Besides, it requires a large training set to train the model. Machine learning methods are suitable for solving multi-classification problems with training samples of corresponding ground-truth labels.

SVM is widely used in various human activity detection and classification systems [12,13,32,37,40,42,44,45,47,48,49,56,59,60,70,72,73,75,76,78,79,83,84,89,90,92,127,130]. SVM classifier are used to classify 10 digital finger gestures [78], eight human movement directions [59], fall detection [37,92], walking direction detection [70], fitness activity classification [56,79], human daily activities [13,40,42,72,75,76,84,90], and human gestures [12,32,73,75,83,89]. Among them, fitness activity mainly contains freehand exercise [56,79] (such as squat, put-up, sit-up, leg-raise, crush, step, etc.). Daily human activities mainly include still, moving, stationary, stand up, sit down, lie down. Human gestures contain rotate, pinch, shake, kick, etc.

The decision tree (DT) can output a simple if-else classification model. With the advantage of less computational cost, it is suitable to solve real-time activity recognition [10,57,85]. The disadvantage is that the correlation between features is ignored, which leads to overfitting. When the number of samples is inconsistent, the decision tree tends to favor the categories with a larger size. APSense [85] uses DT to classify four types of hand motions, which achieves the classification accuracy as high as 90% with 3000 training samples. WiDance [57] yields an overall accuracy of 92% with over 10,000 actions for classifying eight human dance direction through DT.

K-nearest neighbor (KNN) classified by measuring the distance between feature vectors. The disadvantage is that *K* needs to be artificially pre-set. The recognition accuracy may be reduced due to incorrect parameter K settings. Besides, the algorithm is of low sensitivity to outliers. WiSome [59] adopts KNN to classify eight movement directions (front, back, left, right, left-front, right-front, right-back, left-back), which validate an overall recognition accuracy of 95.4% with 5000 records in total. WiFinger [46] applies KNN for nine digital finger gestures (from one to nine) classification. WiFinger acquires 3465 instances and achieves up to 90.4% average classification accuracy. WiChase [40] achieves recognition of three daily activities (running, walking, hand moving) by using KNN. Its classification accuracy is higher than 97% on 720 samples. WiAG [82] recognizes six human gestures (push, pull, flick, circle, throw, dodge) through KNN, which obtains classification accuracy of 91.4% by using 1427 samples from 10 volunteers as a training set.

K-means is an unsupervised learning algorithm without ground truth labels. K-means put similar objects into the same cluster automatically and make messy data becomes organized after K clustering. For example, E-eyes [81] applies K-Means clustering to classify different activities based on the EMD value between CSI samples. The experimental results of clustering achieve over 96% average true positive and less than 1% average false positive for eight activities, which involves 400 CSI sample sets with each set containing 40 CSI samples.

Naive Bayes (NB) requires a few parameters, which are not sensitive to the missing data problem [85]. NB has better precision for the small-scale sample set. The disadvantage is that it can only classify on the assumption that the target characteristics are independent of each other. For example, ApSense [85] uses NB to classify four types of hand motions.

The hidden Markov model (HMM) estimates the joint probability distribution and calculates the posterior probability, which statistically represents the relationship between features and states [36]. It has the advantages of flexibility for dynamic time series. For example, CARM [36] system utilizes the characteristics of the different speeds of the human body in a movement to construct an HMM for each activity with multiple motion states. CARM can classify eight daily human activities (running, walking, sitting down, falling, boxing, brushing teeth, etc.) through HMM. CARM achieves an average accuracy of greater than 96% with 1,400 training samples.

The sparse matrix representation indicates that almost all raw signals can be represented by a linear combination of fewer basic signals. These basic signals, called atoms, are selected from an over-completed dictionary. The elements with the non-zero coefficient in the sparse matrix reveal the main characteristics and intrinsic structure of the signal. The closer the value of the non-zero factor to 1, the higher the signal similarity. The sparse matrix representation can be applied to motion recognition. For example, HeadScan [43] achieves the classification of five daily human activities (coughing, drinking, eating, speaking, and idle) by constructing a sparse matrix via ℓ1 minimization. HeadScan achieves the activity classification accuracy of 86.3% by training through six datasets (2520 training samples).

### 7.3. Deep Learning

Deep learning combines feature extraction and classification to achieve multi-classification of actions. Compared to the machine learning-based method, deep learning requires more training data to determine a large number of parameters. Deep learning does not need feature extraction phases. Guo et al. [88] use DNN to classify ten fitness actions with 3013 training samples, including standing biceps curl, lateral raise, dumbbell curl, leg stretch, pile squat, raise and squat, Tai Chi, dumbbell triceps extension, leg press, and body extension, which achieves an accuracy of 93%.

Convolutional neural network (CNN) is a typical kind of DNN, whose neurons in the neighbor layers are connected through the convolution kernel as an intermediary. CNN has the characteristic of limiting the number of parameters and minimum local structures. Zhang et al. [108] apply CNN to differentiate sit-up, push-up, and walk-out easily. It uses 1980 activities in training and achieves an accuracy of 82%. Sign-Fi [21] adopts nine-layer CNN for 150 gestures of sign language classification. The average recognition accuracy of SignFi is 86.66% for 7,500 instances of 150 sign gestures performed by five volunteers. In order to achieve the environment-independent human motion recognition, EI [77] collects the activities set of 40 subject-room pairs (about 1,200 in total) to train a CNN classifier for six human daily activities sensing (wiping the whiteboard, walking, moving a suitcase, rotating the chair, sitting, standing up and sitting down). Its classification accuracy is between 61–75%.

RNN (recurrent neural network) addresses the limitation of time series on CNN that cannot be modeled. LSTM (long short-term memory) is a typical kind of RNN, which solves the long-term dependency problem. Wi-Multi [97] proposes a deep learning network structure based on LSTM with 936 CSI samples as training data, which achieves a classification accuracy of 96.1%.

Moreover, DFL [95] adopts a sparse autoencoder network with three layers and SoftMax regression to learn features and classify bow, stand, walk, swing, hand clap, etc. DFL achieves 85% accuracy by recording six training sets (1,162 samples) for each activity that performs at 11 locations. Wang et al. [96] develop the self-organizing map network to classify eight fitness activities, including standstill, bow, swing arms, walk, arm up and down, arm left/right, and hand clap. Their system achieves an accuracy of more than 85% by adopting 14 training sets (about 1,680 samples) for training.

## 8. Applications of Wireless Sensing

This survey divides human motion sensing into four types of applications, including detection, recognition, estimation, and tracking.

### 8.1. Detection Applications

The human motion detection can be further divided into fall detection [37,50,73,92], walking step detection [87], intrusion detection [48,49,54,98], and human activity detection [38,72,90,134]. For fall detection, it distinguishes between fall and non-fall activities. Step detection only needs to identify steps and non-steps. For intrusion detection, it discovers a human motion from the received signals. For human activity detection, it is only necessary to distinguish whether human activity happens, instead of distinguishing the types of these activities. Therefore, detection applications are coarse-grained human activity recognitions. Table 9 shows a summary of detection applications based on wireless sensing.

### 8.2. Recognition Applications

Human activity recognitions, can be further divided into hand/finger gesture recognition [14,19,20,39,44,46,47,61,63,69,75,78,85,111,135], limb gesture recognition [16,55,62,64,75,80,82], daily activity recognition [12,13,36,42,43,47,75,76,77,81,84,91,95,96,97,99,123,128], fitness activity recognition [10,56,74,79,84,88], human movement direction recognition [57,59], mouth movement recognition [93], driving gestures [51,109], and fatigue driving posture recognition [45]. Moreover, some research achieves multi-user motion recognition [69,88,97,123]. Table 10 provides the summary of wireless sensing for recognition applications.

Finger gesture recognition is a fine-grained recognition, which requires capturing tiny finger movement variation and accurately distinguish these different subtle change patterns. Finger gesture mainly contain digital finger gesture [44,46,69], directional finger/hand gesture [19,20,39,47,75,78,85] and sign language gesture [21]. Among them, digital gesture recognition usually contains numbers from 1–10. Directional gesture recognition mainly includes simple directional gestures (left, right, push, pull, open, close, up, down, etc.) [19,39,47,75,78,85] and complex directional gestures [20,39,75] (zoom out, zoom in, circle left, circle right, swipe left, swipe right, flip-up, flip-down). Limb gestures mainly include push, dodge, strike, pull, drag, kick, circle, punch twice, and bowl.

The daily human activity contains actions that usually have running, walking, hand moving, bend, phone call, drinking, eating, typing, sit down, and so on. Human movement directions mainly contain left, right, front, back, left-back, left-front, right-back, and right-front. Fitness actions recognition can be further divided into dumbbell exercises [10,74,88] (dumbbell curl, dumbbell triceps extension, dumbbell lift, etc.) and freehand exercises [56,74,79,88,108] (squat, sit-up, push-up, leg-raise, stoop-down, kick, etc.). Compared to finger gesture recognition, fitness action, human gesture, and daily human activity have a larger variation in motion.

### 8.3. Estimation Applications

Estimation application refers to a system that can count the number of actions/steps after activity recognition or activity detection. Estimation applications can be divided into walking step counting [87], fitness motion counting [10,56,79,88,108], and running step counting [86]. Furthermore, it also includes human counting [52,94,125,126,127,130], which contributes to public space management, safety management, energy management, etc. Counting the number of walking is important to ensure and motivate people to exercise every day. Because the purpose of fitness is to strengthen the body, it is essential to ensure a sufficient amount of exercise, so motion counting is required for fitness tracking systems. Fitness activity counting includes a count on the number of fitness action groups and a count on each group of repetitive actions. These fitness exercises can be divided into dumbbell exercises and freehand exercises. Dumbbell movements [10,88] mainly include dumbbell curl, dumbbell triceps extension, dumbbell lift, etc. Dumbbell fitness can install tags on dumbbells to achieve counting of movements [10]. Freehand fitness exercises [56,79,88,108] mainly include squat, sit-up, push-up, leg-raise, leg-press, triceps press, front raise, bent-over row, bicep curl, stoop-down, kick, etc. Table 11 gives a summary of estimation applications based on wireless sensing.

### 8.4. Tracking Applications

For tracking applications, this survey mainly includes human motion tracking [41,71,129], human tracking [15,17,22,24,26,28,29,48,53,58,64,65,66,67,70,100,103,105,110,112,113,128,129,136], in-air finger tracking [11,115], and walking direction tracking [106], and human indoor localization [25,101,102,103,104,114,124,128,132,137,138]. The difference between tracking application and the above three types of applications is that they calculate spatial information, such as direction, speed, or distance estimation. Moreover, it tracks the human for a longer period than the other categories of applications. RF-HMS, an RFID-based human motion-sensing technology, is presented to track device-free human motion through walls [139]. It constructs transfer functions of multipath channel based on phase and RSSI measurements to eliminate device noise and reflections off static objects like walls and furniture without learning the environment of the empty room before. Bekkali et al. [137] introduce a new positioning algorithm for RFID tags using two mobile RFID readers and landmarks, which are passive or active tags with known locations and distributed randomly. This algorithm is based on RSS measurement to calculate the reader-tags distance and target-landmarks distance to estimate the target location.

PinIt [140] is a fine-grained RFID positioning system that works in the absence of a line-of-sight path and the presence of rich multipath. PinIt exploits a tag’s multipath profile to locate it. In particular, signals of nearby tags propagate along closer paths when being reflected off each surface. PinIt obtains a description of all the paths along which a tag’s signal propagates, so its neighboring tags can be identified. The summary of wireless tracking applications is presented in Table 12.

## 9. Challenges and Future Trends of Wireless Sensing

This section presents the challenges and future trends for both current and future human activity sensing solutions with wireless signals.

### 9.1. Wireless Sensing Challenges

From the discussion on the theoretical model and signal processing process, the existing research shares the following common challenges.

Robustness: All the theoretical models are based on the multipath analysis to sift out the motion impact on the received signals. Most research add limitations to the experimental environment to analyze the motion impact through multipath analysis. First, there should not be other persons or moving objects around. Other people or moving object’s actions are also captured by the received signals, which makes it hard to sift the target person’s action effect. Second, the action performer is often needed to be on a fixed position from the sender and receiver of the signal in advance for learning-based methods. Otherwise, the learning model would fail to detect or recognize activities. Third, there are often some specific areas for activity recognition. For the Fresnel Zone model, most research takes place at the boundary on the first 8–12 FFZs in deployment. However, it is ridiculous for users to calculate this specific place in reality. When targeting the real scenarios of wireless motion sensing, all the above limitations should be eliminated. It may require some new theoretical models to construct relations between human motion and wireless signals or some novel signal processing methods.

Non-coexistence of sensing and communications: Wireless infrastructures are designed for signal communications, not for sensing applications. The existing approaches require deploying and controlling both the sender and receiver of the wireless infrastructure. Some sensing applications even require a high frequency of continuous sinusoid signals to achieve high performance. This adds the burden to the scarce bandwidth resources and results in reduced communication performance and efficiency. Moreover, sending the continuous sinusoid signals also affect the communications between nearby wireless devices.

Potential privacy threat: Wireless activity sensing takes advantage of non-intrusive and non-obtrusive. However, it still introduces some privacy concerns. As shown in Section 8, existing researches have been able to sense and estimate some daily activities and fitness activity by indoor wireless infrastructure. Such information can be leaked to malicious attackers when the victim may be unaware of the existence of wireless sensing. This imposes a conflict with the robustness issue, which targets on improve sensing under various scenarios. So new techniques or algorithms are needed to ensure users’ right to know and control the wireless sensing systems, especially the receivers beforehand.

Multiple user activity sensing: Wireless signals are sensitive to any movements in the sensing area, because any motions may change the multipath propagation of the wireless signals. When multiple persons are sharing the same physical space, the received signals will contain all the impacts by all the persons’ motions. Existing FMCW and antenna array-based solutions like [17] can track the hand gestures of multiple people simultaneously, leveraging a directional antenna array. For other wireless signals, a promising way to isolate concurrent activities of different people is to separate sensing space with a complex web of wireless links in the area. Nevertheless, it is still challenging to address the effect of multiple users and recognize different actions conducted by different users with a limited number of wireless links.

Limited sensing range: Although multiple types of wireless signals can be used in human activity sensing, the sensing range is still limited. For example, acoustic-based sensing has a sensing range of 1–2 m, while RFID and WiFi have a sensing range of 2–8 m. The sensing range of VLC is 3–7 m. While LoRa signals have a communication range of 10 km, the current sensing range is below 100 m. Moreover, the applicable sensing systems are still lacking for outdoor environments due to the limited sensing range.

Complex deep learning: Some CSI-based activity recognition applications exploit deep learning approaches, for they can automatically extract high-level features from CSI streams for classification. The deep learning approaches, however, require not only an extensive training set to train the underlying parameters of the learning network and but also a comparable computation and storage capacity to perform training. Therefore, it adds to the burden on the users to collect training samples and maybe not computable on resources limited devices such as wearable and edge devices.

Lack of standard datasets: Currently, most wireless activity sensing studies evaluate their performance using their dataset. Researchers have to recruit some volunteers to conduct many types of actions to collect wireless signal streams. Moreover, the experimental environments are often chosen according to the particular targets of the applications. Consequently, the system performance often depends on the deployment and the collection process, which makes it difficult for comparison among different studies.

### 9.2. Feature Trends in Wireless Sensing

This section presents future trends in addressing the above challenges and issues.

New theoretical models: The existing model concentrates on the reflection of the human body to the signal propagation, which is captured through the multipath effect. The signal reflection from the human body to the receiver often requires specific positions and angles. It imposes the limitation on the application environment. For example, if the action is extracted from the received signals through the signal diffraction model, the restriction of the specific position may be eliminated. As long as the human performs activities close to the receiver, it will create the diffraction effect with similar patterns inside the received signals. The diffraction model may solve the robust challenge as the diffraction effect depends little on the objects at a certain distance away. If every user has her/his wireless signal receiver, the diffraction effect will naturally separate the sensing space for each user, which also solves the challenge of multiple users. Moreover, a new theoretical model will guide the activity classification process, which may eliminate or reduce the complexity of applying deep learning.

Coexistence of sensing and communications: The major obstacle for the coexistence of sensing and communication is that current solutions need to control the sender of wireless infrastructure and require specific continuous signals for sensing. If the wireless signals already in space can be directly for sensing, the sensing system may only focus on listening and does not need to control the sender of infrastructure. Then the coexistence of sensing and communications can be realized. Moreover, a wireless sensing solution with only receivers may apply the mobile signal infrastructure, which has the advantage of ubiquitous coverage and tackles the sensing range limit.

Awareness of sensing on receivers: The privacy concerns come from that specific systems may make use of some indicators of the received signals for sensing purposes. The tools to control and report on the usage of received signals except communication are of importance. Moreover, more research efforts should be concentrating on the signal receiver of smartphones. The reason is two-fold. Firstly, smartphones are ubiquitous receivers as people carry them all the time. Secondly, users are familiar with the privacy control procedure with smartphones [141], so the signal usage tool to control privacy can be quickly adopted by the users.

Constructing open datasets: It is still an open question to construct the standard datasets for wireless sensing research. When constructing open datasets, many factors have to be carefully chosen, including test environments, deployment of wireless transceivers, types of wireless signals, number of volunteers, differences among volunteers, action types, and size of samples. An open standard dataset will help accelerate the wireless sensing study and improve performance evaluation and comparison.

According to the directions mentioned above, more research efforts should be put on the wireless sensing solution with just a smartphone as the receiver, which directly makes use of ubiquitous mobile signals for sensing under the guidance of a new theoretical model between human motion and wireless signals.

## 10. Conclusions

This survey gives a comprehensive review of the background of wireless signals, the theoretical models from wireless signals to human actions, signal pre-processing techniques, signal segmentation techniques, feature extraction, activity recognition, and applications of wireless sensing. The article highlights seven wireless sensing challenges on human activities: robustness, non-coexistence of sensing and communications, potential privacy threat, multiple user activity sensing, limited sensing range, complex deep learning, and lack of standard datasets. Finally, the survey points out four future research trends: new theoretical models, the coexistence of sensing and communications, and awareness of sensing on receivers, and constructing open datasets.

## Figures and Tables

**Table 1:** Summary of related surveys on wireless sensing.

Reference	Signals	Topic Focus	Application Scope
Yang et al. [1], 2013	Wi-Fi (RSS, CSI)	RSS and CSI-based solutions	indoor localization
Xiao et al. [2], 2016	UWB, RFID, Wi-Fi, acoustic	models, basic principles, and data fusion techniques	indoor localization
Zou et al. [3], 2017	Wi-Fi (CSI)	model-based (CSI-Speed model, Fresnel zone model) approaches	human behavior recognition
Wu et al. [4], 2017	Wi-Fi (CSI)	pattern-based and model-based (CSI-Speed model, AoA model, Fresnel zone model)	human behavior recognition, respiration detection
Yousefi et al. [5], 2017	Wi-Fi (CSI)	deep learning classification	human behavior recognition
Al-qaness et al. [6], 2019	Wi-Fi (CSI)	CSI-based sensing mechanism, methodology (signal pre-processing, feature extraction, classification), limitations and challenges	detection (motion), recognition (daily activity, hand gesture), localization
Wang et al. [7], 2019	Wi-Fi (CSI)	base signal selection, signal pre-processing feature extraction, classification, issues, future trends	behavior recognition
Ma et al. [8], 2019	Wi-Fi (CSI)	signal processing, model-based and learning-based algorithms, performance, challenges, future trends	detection, recognition, estimation, tracking,
Liu et al. [9], 2019	Wi-Fi (RSSI, CSI), FMCW, Doppler shift	basic principles, techniques and system structures, future directions and limitations	detection, recognition, localization, tracking
This survey	RFID, FMCW, Wi-Fi, visible light, LoRa, acoustic, LTE	model (Doppler, Fresnel zone, FMCW, AoA, mD-Track), signal pre-processing, segmentation, feature extraction, classification, challenges, future trends	detection, recognition, estimation, tracking

**Table 2:** Summary of different types of RFID. Here *p* represents passive and *a* represents active.

Type	Energy	Frequency	Distance	Penetration
LF	p	125 kHz	≤10 cm	blocked by metal
HF	p/a	13.56 MHz	≤1.2 m	blocked by metal
UHF	p/a	860~960 MHz	≤4 m	blocked by metal, liquid
microwave	p/a	2.45 GHz, 5.8 GHz	≤100 m	blocked by metal, liquid

**Table 3:** Comparison between RSS and CSI.

Attribute	RSS	CSI
network layer	MAC	physical
access	communications equipment	CSI tool
generalization	all devices	some devices
sensitivity	low	high
time resolution	packet-scale	multi-path signal cluster scale
frequency resolution	/	subcarrier scale

**Table 4:** Summary of models between human activities and parameters of wireless signal propagation

Signal Feature	Motion Feature	Models
Phase	Velocity	Coarse-grained estimation [13,20,21,26,33,37,38,39,40,41,42,43,44,45,46,47,48,49,50,51,52,53,54]
Frequency	Velocity	Doppler effect model [10,12,22,55,56,57,58,59,60,61,62,63], FMCW chirp model [17,64]
	Direction	Doppler effect model [22,57,58]
	Distance (dRX)	FMCW chirp model [15,16,17,64,65,66,67,68]
Amplitude	Velocity	CSI-Speed model [36,69], Coarse-grained estimation [14,18,19,23,24,25,26,37,40,42,43,46,47,49,50,52,53,54,70,71,72,73,74,75,76,77,78,79,80,81,82,83,84,85,86,87,88,89,90,91,92,93,94,95,96,97,98,99,100,101,102,103,104,105]
	Distance (dLoS)	Fresnel zone model [106,107,108,109,110]
	Direction	Fresnel zone model [106], AoA with antenna array [11,15,65,111,112,113,114,115]
aw Signal	Distance, Direction, Velocity	mD-Track [116]

**Table 5:** Summary of signal preprocessing.

Category	Example	Pros and Cons
Noise Reduction	time-domain filter: moving average [14,45,53,54,70,73,100,123], median [74], single-sideband Gaussian [76], weighted moving average [42,46,73,75,87,103,124], local outlier factor [59], Hampel filter [49,59,77,86], Savitzky–Golay filter [86,108,125,126]	Pros: low computation cost, suitable for coarse-grained motion recognition and tracking; Cons: poor sensitivity to fine-grained gestures
Noise Reduction	frequency-domain filter: passband [37,41,44,57,58,87,93], wavelet [19,59,87,92,93,95,97,127], Kalman [10,24,25,32,41,50,66,103,124], Butterworth [12,19,39,40,43,46,51,52,70,71,74,75,80,81,82,83,89,108], Birge–Massart [78]	Pros: high sensitivity to all activities including finger gestures; Cons: complex calculation.
Calibration	interpolation [37,51,66,92], normalization [50,77,79,94,128,129], phase calibration [21,42,44,51,52,68,90]	
Redundant Information Removal	first PC selected [42,45,57,58,84], first PC discarded [36,80], second PC selected [51], third PC selected [69], first two PCs selected [43], top-5 subcarriers [39], static environment removal [15,22,65], multipath mitigation [20,68,87,93,102,105,110,111,113,114]	

**Table 6:** Summary of signal segmentation.

Category	Type	Pros and Cons	Examples
Time-domain threshold	Phase	Pros: high sensitivity; Cons: poor robustness	phase difference [23,26,37,71]
	Amplitude	Pros: easy to access; Cons: noise variation	amplitude [19,20,82,83,84,86], amplitude difference [14,44], amplitude variance [23,24,40,85,97,108]
	Statistics	Pros: accurate; Cons: complex computation	cumulative moving variance [75,76,81], average RSS [39], movement indicator threshold [36,46,87], CV threshold [45], SVR and LVR [38], LOF anomaly detection [73,76,78]
	Energy	Pros: easy to access; Cons: susceptible to noise	energy [45,89]
	Similarity	Pros: suitable for repetitive motion; Cons: poor tolerance	autocorrelation [82], optimize template and cutting segments alternately [79]
Frequency-domain threshold	Peak	Pros: directly available; Cons: susceptible to noise	spectrum [25,26,28,29,69], Doppler shift [55,56,57,58]
	Energy	Pros: easy to collect; Cons: susceptible to noise	energy [88,89,98,99,105,113]
	Similarity	Pros: suitable for cutting repetitive motion; Cons: poor tolerance	Kullback–Leibler divergence [10], impulse [56]

**Table 7:** Summary of features.

Category	Pros and Cons	Features
Time-domain	Pros: relatively simple calculation; Cons: vulnerable to environmental changes and noise	maximum, minimum, mean, standard deviation, kurtosis, skewness, variance, median and median absolute deviation, percentiles, root sum square, interquartile range [13,18,37,40,42,43,46,48,49,50,62,72,73,75,76,78,79,80,83,84,85,88,89,90,97,108,127,130], time lag [37], power decline ratio [37], amplitude sequence [20,54,74,91], phase sequence [39,51,107], CV [38]
Frequency-domain	Pros: capture the periodical characteristics of human motion; Cons: large amount of calculation	FFT coefficient [12,83], dominant frequency [43], power spectrum density [88], spectral entropy [43,57,70,72,73,80,90,92], fractal dimension [92], frequency domain energy [85], Doppler velocity intensity [56], frequency sequence [10,26,28,29,49,55,60,95,98,104,113,114,124], spectrograms [19,47]
Time-Frequency domain	Pros: reflects both time and frequency domain information; Cons: heavy calculation	DWT coefficient [23,36,42,82,93,97,99,103,123,125,126,131], HHT [45,80]
Spatial domain	Pros: suitable for localization and tracking; Cons: often need specific equipment	AoA [15,22,25,41,65,66,112,113,114,115], distance [15,26,100,105,106,110], ToF [41,64,66,68,113,132]

**Table 8:** Summary of the activity classification.

Category	Pros and Cons	Examples
Template Matching	Pros: no training needed; Cons: accuracy depending on the specific template	Euclidean distance [19,94], DTW [10,20,39,42,55,74,91,93,97], EMD [54,70,81,89], Jaccard coefficients [69]
Machine Learning	Pros: high efficiency and robustness; Cons: a lot of training data required	KNN [18,40,46,48,51,52,59,82,99,124], SVM [12,13,32,37,40,42,44,45,47,48,49,56,59,60,70,72,73,75,76,78,79,83,84,89,90,92,97,127,130], decision tree [10,57,85], K-means [76,81], Naive Bayes [85], HMM [36], sparse representation via ℓ1 minimization [43]
Deep Learning	Pros: strong learning ability and portability; Cons: large amount of training data required	CNN [16,17,21,48,67,77,108,126], DNN [59,88], SOM [96], sparse autoencoder network [95], LSTM [97]

**Table 9:** Summary of wireless sensing applications: detection

Application	Reference	Signal	Model	Signal Processing	Classification
fall detection	WiFall [73], 2014	Wi-Fi (CSI)	amplitude estimation	moving average filter	time domain feature + SVM
fall detection	Anti-fall [50], 2015	Wi-Fi (CSI)	amplitude and phase estimation	low-pass filter, normalization	time domain feature + SVM
fall detection	RT-fall [37], 2017	Wi-Fi (CSI)	amplitude estimation	passband filter, interpolation + phase difference	time domain feature + SVM
fall detection	FallDefi [92], 2018	Wi-Fi (CSI)	amplitude estimation	wavelet filter, interpolation	frequency domain feature + SVM
intrusion detection	Zieger [98], 2009	acoustic	amplitude estimation	frequency quantity	/
intrusion detection	DeMan [49], 2015	Wi-Fi (CSI)	amplitude and phase estimation	Hampel filter, linear fitting	time and frequency domain feature + SVM
motion detection	FRID [38], 2015	Wi-Fi (CSI)	phase estimation	SVR and LVR threshold	/
motion detection	Liu et al. [72], 2017	Wi-Fi (CSI)	amplitude estimation	segmentation by skewness	time and frequency domain feature + SVM
walking step detection, counting	WiStep [87], 2018	Wi-Fi (CSI)	amplitude estimation	passband filter wavelet filter, weighted moving average, multipath mitigation	/
walking direction detection, respiration rate detection	Zhang et al. [107], 2017	Wi-Fi (CSI)	Fresnel zone model	multiple carrier frequencies	/

**Table 10:** Summary of wireless sensing applications: recognition.

Application	Reference	Signal	Model	Signal Processing	Classification
fitness activity recognition and counting	FEMO [10], 2017	RFID	Doppler effect model	Kalman filter + Kullback–Leibler divergence	frequency sequence feature + decision tree, DTW
fitness activity recognition, user identification	Guo et al. [88], 2018	Wi-Fi (CSI)	amplitude estimation	low-order polynomial fitting + subtract mean value	DNN
fitness activity recognition, counting	WiFit [56], 2018	Wi-Fi (CSI)	Doppler effect model	Doppler frequency shift peak threshold	frequency-domain feature + SVM
fitness activity recognition, counting	Motion-Fi [79], 2018	RFID	amplitude estimation	normalization + optimize template, cut segments alternately	time domain feature + SVM
fitness activity recognition, counting	Zhang et al. [108], 2019	Wi-Fi (CSI)	Fresnel zone model	Savitzky–Golay filter + amplitude variance threshold	time domain feature + CNN
fitness activity recognition	SEARE [74], 2019	Wi-Fi (CSI)	amplitude estimation	Butterworth low-pass filter, median filter	time domain feature + DTW
daily activity recognition	Kim et al. [60], 2009	Doppler radar	Doppler effect model	noise threshold filtering based on Gaussian distribution	frequency domain feature + SVM/DT
daily activity recognition	Sekine et al. [12], 2012	Doppler sensor	Doppler effect model	Butterworth low-pass filter	frequency domain feature + SVM
daily activity recognition	Sigg et al. [99], 2013	RF signal (RSSI)	RSSI fingerprints model	normalized spectral energy	time-frequency domain feature + template matching
daily activity recognition	Sigg et al. [18], 2013	Wi-Fi (CSI)	amplitude estimation	/	time domain feature + KNN
daily activity recognition	E-eyes [81], 2014	Wi-Fi (CSI)	amplitude estimation	Butterworth low-pass filter + cumulative moving variance threshold	time domain feature + EMD/ K-means
daily activity recognition	IDSense [12], 2015	RFID	phase estimation	2-s sliding window	time domain feature + SVM
daily activity recognition	Wang et al. [76], 2015	Wi-Fi (CSI)	amplitude estimation	single-sideband Gaussian filter+ LOF Anomaly Detection	time domain feature + SVM
daily activity recognition	CARM [36], 2015	Wi-Fi (CSI)	CSI-Speed model	first PC discard + movement indicator threshold	DWT feature + HMM
daily activity recognition	Headscan [43], 2016	Wi-Fi (CSI)	amplitude estimation	Butterworth low-pass filter, first two PC selection	time and frequency domain feature + sparse representation via ℓ_1_ minimization
daily activity recognition	Mudra [91], 2016	Wi-Fi (CSI)	amplitude estimation	finite impulse	time-domain feature + DTW
daily activity recognition	BodyScan [84], 2016	Wi-Fi (CSI)	amplitude estimation	first PC selection + amplitude threshold	time domain feature + SVM
daily activity recognition	DFL [95], 2017	Zigbee RSS	amplitude estimation	wavelet filter	sparse autoencoder network
daily activity recognition	PeriFi [90], 2017	Wi-Fi (CSI)	amplitude estimation	phase calibration	time and frequency domain feature + SVM
daily activity recognition	WiChase [40], 2017	Wi-Fi (CSI)	amplitude and phase estimation	Butterworth low-pass filter + amplitude variance threshold	time domain feature + KNN, SVM
daily activity recognition	EI [77], 2018	Wi-Fi, ultrasound	amplitude estimation	Hampel filter, normalization	CNN
daily activity recognition	Wang et al. [96], 2018	Wi-Fi (CSI)	amplitude estimation	median filter, linear	SOM
daily activity recognition	HuAc [75], 2018	Wi-Fi (CSI)	amplitude estimation	Butterworth filter, weighted moving average + moving variance threshold	time domain feature + SVM
daily activity recognition	WiMotion [42], 2019	Wi-Fi (CSI)	amplitude and phase estimation	weighted moving average, phase calibration, first PC selection	time domain feature + DTW, SVM
daily activity recognition	Wi-Multi [97], 2019	Wi-Fi (CSI)	amplitude estimation	PCA, DWT + amplitude variance	time and frequency domain + LSTM
daily activity recognition	MultiTrack [123], 2019	Wi-Fi (CSI)	amplitude estimation	DWT + moving average filter	time and frequency domain+ DTW
moving direction recognition	WiDance [57], 2017	Wi-Fi (CSI)	Doppler effect model	first PC selection + peak threshold of Doppler frequency shift	frequency domain feature + DTW
moving direction recognition	WiSome [59], 2017	Wi-Fi (CSI)	Doppler effect model	local outlier factor, wavelet filter	frequency domain feature + DNN, SVM
sign language gesture recognition	SignFi [21], 2018	Wi-Fi (CSI)	phase estimation	phase calibration	CNN
limb gesture recognition	Humanten-na [83], 2012	wireless	amplitude estimation	Butterworth low-pass filter + amplitude threshold	time and frequency domain feature + SVM
limb gesture recognition	WiSee [55], 2013	Wi-Fi (CSI)	Doppler effect model	Doppler frequency shift threshold	frequency domain feature + DTW
limb gesture recognition	Soli [62], 2016	FMCW	Doppler effect model	soli processing pipeline + high temporal resolution	time-frequency domain feature + random forest, Bayesian network
limb gesture recognition	WIAG [82], 2017	Wi-Fi (CSI)	amplitude estimation	Butterworth filter + amplitude threshold	DWT feature + KNN
limb gesture recognition	Mohamm-ed et al. [80], 2019	Wi-Fi (CSI)	amplitude estimation	Butterworth, first PC discard	time domain feature + random forest
coarse gesture estimation	RF-Pose [16], 2018	FMCW	FMCW chirp model	spectrogram	CNN
finger/hand ~gesture recognition	Kalgaonkar et al. [63], 2009	ultrasonic	Doppler effect model	downsampling + PCA	Gaussian mixture model + Bayesian
finger/hand ~gesture recognition	Melgarejo et al. [39], 2014	directional antenna	phase estimation	Butterworth low-pass filter, 5 top subcarriers selection + average RSS threshold	time domain feature + DTW
finger/hand gesture recognition	Apsense [85], 2014	Wi-Fi (CSI)	amplitude estimation	amplitude variance threshold	time and frequency domain feature + decision tree, naive Bayes
finger/hand gesture recognition	AllSee [14], 2014	RFID	amplitude estimation	moving average filter + amplitude difference threshold	time domain feature + template matching
finger/hand gesture recognition	Molchanov et al. [61], 2015	FMCW	Doppler effect model	static background subtraction	frequency domain feature + template matching
finger/hand gesture recognition	WiGest [19], 2015	Wi-Fi (CSI)	amplitude estimation	Butterworth low-pass filter, wavelet filter + amplitude threshold	time domain feature + template matching
finger/hand gesture recognition	WiG [78], 2015	Wi-Fi (CSI)	amplitude estimation	Birge–Massart filter + LOF Anomaly Detection	time domain feature + SVM
finger/hand gesture recognition	Demum [44], 2016	Wi-Fi (CSI)	phase estimation	passband filter, phase calibration + amplitude difference	time domain feature + SVM
finger/hand gesture recognition	Tan et al. [20], 2016	Wi-Fi (CSI)	phase estimation	multipath mitigation + amplitude threshold	time domain feature + DTW
finger/hand gesture recognition	Li et al. [46], 2016	Wi-Fi (CSI)	amplitude and phase estimation	Butterworth filter, weighted moving average + movement indicator threshold	time domain feature + KNN
finger/hand gesture recognition	DELAR [47], 2017	Wi-Fi (CSI)	amplitude and phase estimation	phase and amplitude threshold	heat map + DNN
finger/hand gesture recognition	WIMU [69], 2018	Wi-Fi (CSI)	CSI-Speed model	third PC selection + frequency quantity threshold	frequency domain feature + Jaccard coefficients
finger/hand gesture recognition	WiCatch [111], 2018	Wi-Fi (CSI)	AoA model with antenna array	multipath mitigation	spectrum feature + SVM
fatigue driving posture recognition	WiFind [45], 2018	Wi-Fi (CSI)	phase estimation	moving average filter, first PC selection + CV threshold	HHT feature + SVM
driving gestures recognition	WiTraffic [89], 2017	Wi-Fi (CSI)	amplitude estimation	Butterworth low-pass filter + energy threshold	time domain feature + SVM/EMD
driving gestures recognition	WiBot [51], 2018	Wi-Fi (CSI)	phase estimation	Butterworth low-pass filter, interpolation, phase calibration, second PC selection + impulse window detection	time domain feature + KNN
driving Gestures recognition	WiDriver [109], 2018	Wi-Fi (CSI)	Fresnel zone model	subcarrier selection	finite automata model + BP
mouth movement recognition	WiHear [93], 2014	Wi-Fi (CSI)	amplitude estimation	passband filter, wavelet filter, multipath mitigation	DWT feature + DTW

**Table 11:** Summary of wireless sensing applications: estimation.

Application	Reference	Signal	Model	Signal Processing	Classification
fitness activity recognition, counting	FEMO [10], 2017	RFID	Doppler effect model	Kalman filter + Kullback–Leibler divergence	frequency domain feature + DTW, decision tree
fitness activity recognition, counting	WiFit [56], 2018	Wi-Fi (CSI)	Doppler effect model	Doppler frequency shift peak threshold	frequency domain feature + SVM
fitness activity recognition, counting	Motion-Fi [79], 2018	RFID	amplitude estimation	normalization + optimize template, cut segments alternately	time domain feature + SVM
fitness activity recognition, counting, user identification	Guo et al. [88], 2018	Wi-Fi (CSI)	amplitude estimation	low-order polynomial fitting + subtract	DNN
fitness activity recognition, counting	Zhang et al. [108], 2019	Wi-Fi (CSI)	Fresnel zone model	Savitzky–Golay filter + amplitude variance threshold	time domain feature + CNN
walking step detection, counting	WiStep [87], 2018	Wi-Fi (CSI)	amplitude estimation	passband filter + wavelet filter + weighted moving average + multipath mitigation	/
running step counting	Wi-Run [86], 2018	Wi-Fi (CSI)	amplitude estimation	Savitzky–Golay filter, Hampel filter + amplitude threshold	time domain feature+ Frechet distance
human counting	FCC [94], 2014	Wi-Fi (CSI)	Grey Verhulst model	percentage of zero elements	time domain feature + SVM
human counting	Domenico et al. [52], 2016	Wi-Fi (CSI)	amplitude estimation	normalization	time domain feature+ Euclidean distance, linear discriminant classifier
human counting	MAIS [130], 2017	Wi-Fi (CSI)	amplitude and phase estimation	low pass filter, phase calibration	time domain feature + KNN
human counting	FreeCount [127], 2017	Wi-Fi (CSI)	phase estimation	wavelet-based filter	time domain feature + SVM
human counting	Wi-Count [125], 2018	Wi-Fi (CSI)	phase estimation	Savitzky–Golay filter, amplitude threshold	time and frequency domain feature + K-means
human counting	Door-Monitor [126], 2019	Wi-Fi (CSI)	amplitude and phase estimation	Savitzky–Golay filter, phase calibration	time domain feature + CNN

**Table 12:** Summary of wireless sensing applications: tracking.

Application	Reference	Signal	Model	Signal Processing	Localization Feature
human tracking	Youssef et al. [100], 2007	Wi-Fi (RSSI)	RSSI estimation	moving average filter + RSSI threshold	distance feature
human tracking	Feger et al. [65], 2009	FMCW	FMCW chirp model + AoA with antenna array	static environment partial removal	AoA in spatial domain feature
human indoor localization, tracking	SPKS [103], 2009	Wi-Fi (RSSI)	amplitude estimation	Kalman filter + weighted moving average threshold	RSSI maps incorporated into a Bayesian framework
human tracking	Wilson et al. [128], 2010	RF signal (RSSI)	radio tomograph-ic imaging	normalization, weighted threshold	spatial covariance feature
human tracking	Gierlich et al. [67], 2011	FMCW	FMCW chirp model	spectrogram	CNN
human tracking	VRTI [129], 2011	RF signal (RSSI)	radio tomograph-y	Kalman filter, normalization	radio tomographic imaging feature
human tracking	FILA [110], 2012	Wi-Fi (CSI)	Fresnel zone model	multipath mitigation	distance feature
human tracking	WiVi [112], 2013	Wi-Fi	AoA with antenna array	initial nulling	AoA in spatial domain feature
human tracking	WiTrack [66], 2013	FMCW	FMCW chirp model	Kalman filter + interpolation	ToF, AoA feature
human tracking	Pilot [53], 2013	Wi-Fi (CSI)	amplitude and phase estimation	moving average filter RSSI threshold	time and frequency domain feature + fingerprinting
human tracking	Zhou et al. [54], 2013	Wi-Fi (RSSI)	amplitude and phase estimation	low-pass filter, moving average filter	time domain feature+ EMD
human tracking	Wang et al. [105], 2013	Zigbee RSS	amplitude estimation	multipath mitigation + frequency-domain threshold	distance feature
human tracking	WIZ [64], 2014	FMCW	FMCW chirp model	map them into 2D heatmaps	ToF feature
human tracking	RF-Capture [15], 2015	FMCW	FMCW chirp model + AoA with antenna array	static environment partial removal	AoA, distance in spatial domain feature
human tracking	LiSense [24], 2015	VLC	amplitude estimation	Kalman filter + amplitude variance + frequency shift	human skeleton feature
human tracking	WiTrack2.0 [68], 2015	FMCW	FMCW chirp model	multipath mitigation, phase calibration	ToF feature
human tracking	IndoTrack [22], 2017	Wi-Fi (CSI)	Doppler effect model	static environment partial removal	AoA in spatial domain feature
human tracking	Widar [58], 2017	Wi-Fi (CSI)	Doppler effect model	passband filter + first PC selection + peak threshold + Doppler frequency shift	frequency domain feature+ searching with least fitting error
human tracking	Guo et al. [70], 2017	Wi-Fi (CSI)	amplitude estimation	moving average filter, Butterworth	time and frequency domain feature + EMD, SVM
human tracking	Backscatt-er [28], 2017	LoRa	reconfigurable antenna model	Doppler frequency shift	/
human tracking	Strata et al. [26], 2017	acoustic	amplitude and phase estimation	frequency quantity, phase difference	distance feature
human tracking	PhaseMo-de [48], 2018	Wi-Fi (CSI)	phase estimation	median filter + phase threshold	time domain feature + SVM, random forest, KNN
human tracking	Karanam et al. [113], 2019	Wi-Fi (CSI)	AoA model with antenna array	multipath mitigation	AoA, ToF
human tracking	Chan et al. [17], 2019	FMCW	FMCW chirp model	spectrogram	CNN
human tracking	WideSee [29], 2019	LoRa	reconfigurable antenna model	Doppler frequency shift	direction-related feature
human indoor localization	RADAR [102], 2000	RF signal	amplitude estimation	multipath mitigation	frequency-domain feature + K-means
human indoor localization	EZ [104], 2010	Wi-Fi (RSSI)	amplitude estimation	/	time domain feature + resolution generation algorithm
human indoor localization	PinLoc [101], 2012	Wi-Fi (CSI)	amplitude estimation	divergence	frequency-domain feature + K-means
human indoor localization	CUPID [114], 2013	Wi-Fi (CSI)	AoA with antenna array	multipath mitigation	AoA feature
human indoor localization	Epsilon [25], 2014	VLC	amplitude estimation	Kalman filter + frequency quantity threshold	AoA feature
human indoor localization	TCPF [124], 2015	Wi-Fi (RSSI)	amplitude estimation	Kalman filter, weighted moving average	Frequency-domain feature + KNN
human localization	Chronos [132], 2016	Wi-Fi (CSI)	amplitude estimation	packet detection delay removal, multi-path separation	ToF feature
human motion tracking	WiDeo [41], 2015	Wi-Fi (CSI)	phase estimation	Kalman filter	AoA, ToF feature
human motion tracking	MoSense [71], 2017	RF signal	amplitude estimation	Butterworth low-pass filter + phase difference threshold	time domain feature + binary classification
in-air hand tracking	RF-IDraw [10], 2014	RFID	AoA with antenna array	/	multi-resolution positioning algorithm
in-air hand tracking	WiDraw [115], 2015	Wi-Fi (CSI)	AoA with antenna array	low pass filter	AoA feature
walking direction tracking	WiDir [106], 2016	Wi-Fi (CSI)	Fresnel zone mode	cross-correlation denoising, polynomial smoothing filter + angle threshold	spatial and time domain feature
/	Minh [23], 2009	VLC	amplitude estimation	phase difference + amplitude variance	/
